# Proteomics profiling of vitreous humor reveals complement and coagulation components, adhesion factors, and neurodegeneration markers as discriminatory biomarkers of vitreoretinal eye diseases

**DOI:** 10.3389/fimmu.2023.1107295

**Published:** 2023-02-16

**Authors:** Fátima M. Santos, Sergio Ciordia, Joana Mesquita, Carla Cruz, João Paulo Castro e Sousa, Luís A. Passarinha, Cândida T. Tomaz, Alberto Paradela

**Affiliations:** ^1^ CICS-UBI – Health Sciences Research Centre, University of Beira Interior, Covilhã, Portugal; ^2^ Functional Proteomics Laboratory, Centro Nacional de Biotecnología, CSIC, Madrid, Spain; ^3^ Chemistry Department, Faculty of Sciences, University of Beira Interior, Covilhã, Portugal; ^4^ Department of Ophthalmology, Centro Hospitalar de Leiria, Leiria, Portugal; ^5^ Associate Laboratory i4HB - Institute for Health and Bioeconomy, Faculdade de Ciências e Tecnologia, Universidade NOVA, Caparica, Portugal; ^6^ UCIBIO–Applied Molecular Biosciences Unit, Departamento de Química/Departamento Ciências da Vida, Faculdade de Ciências e Tecnologia, Universidade NOVA de Lisboa, Caparica, Portugal; ^7^ Laboratório de Fármaco-Toxicologia, UBIMedical, Universidade da Beira Interior, Covilhã, Portugal

**Keywords:** age-related macular degeneration, biomarkers, complement and coagulation cascades, extracellular matrix, neurodegeneration, proliferative diabetic retinopathy, retinal detachment, vitreous proteomics

## Abstract

**Introduction:**

Diabetic retinopathy (DR) and age-related macular degeneration (AMD) are leading causes of visual impairment and blindness in people aged 50 years or older in middle-income and industrialized countries. Anti-VEGF therapies have improved the management of neovascular AMD (nAMD) and proliferative DR (PDR), no treatment options exist for the highly prevalent dry form of AMD.

**Methods:**

To unravel the biological processes underlying these pathologies and to find new potential biomarkers, a label-free quantitative (LFQ) method was applied to analyze the vitreous proteome in PDR (n=4), AMD (n=4) compared to idiopathic epiretinal membranes (ERM) (n=4).

**Results and discussion:**

Post-hoc tests revealed 96 proteins capable of differentiating among the different groups, whereas 118 proteins were found differentially regulated in PDR compared to ERM and 95 proteins in PDR compared to dry AMD. Pathway analysis indicates that mediators of complement, coagulation cascades and acute phase responses are enriched in PDR vitreous, whilst proteins highly correlated to the extracellular matrix (ECM) organization, platelet degranulation, lysosomal degradation, cell adhesion, and central nervous system development were found underexpressed. According to these results, 35 proteins were selected and monitored by MRM (multiple reaction monitoring) in a larger cohort of patients with ERM (n=21), DR/PDR (n=20), AMD (n=11), and retinal detachment (n=13). Of these, 26 proteins could differentiate between these vitreoretinal diseases. Based on Partial least squares discriminant and multivariate exploratory receiver operating characteristic (ROC) analyses, a panel of 15 discriminatory biomarkers was defined, which includes complement and coagulation components (complement C2 and prothrombin), acute-phase mediators (alpha-1-antichymotrypsin), adhesion molecules (e.g., myocilin, galectin-3-binding protein), ECM components (opticin), and neurodegeneration biomarkers (beta-amyloid, amyloid-like protein 2).

## Introduction

Despite improvements in the prevention and control of ocular diseases in the past 30 years, the public health burden associated to visual impairment and blindness is expected to increase due to the growth and aging of the world population and the increase in the prevalence of chronic diseases such as diabetes mellitus (1, 2). Diabetic retinopathy (DR) and age-related macular degeneration (AMD) are leading causes of visual impairment and blindness in middle-income and industrialized countries and, therefore, they are considered priority eye diseases by World Health Organization (3). DR is a microvascular complication that develops in patients with diabetes (4). In the non-proliferative stage, microvascular changes such as microaneurysms, basement membrane thickening, and loss of pericytes occur in response to hyperglycemia, but increasing evidence suggests that microvascular changes may be preceded by neuroglial degeneration. Proliferative diabetic retinopathy (PDR) is led by retinal ischemia, which combined with an imbalance in the levels of inflammatory and pro-angiogenic cytokines can trigger intra-retinal and intravitreal neovascularization (NV) (1, 4, 5). AMD is a multifactorial disease characterized by the loss of central vision due to the degeneration of photoreceptors and retinal pigment epithelium (RPE) (6, 7). In an early phase, AMD is characterized by the accumulation of yellowish deposits (drusen) underneath the retina but it can further progress to late AMD, recognized either by progressive atrophy of RPE (Dry AMD), non-neovascular intraretinal exudation, or macular neovascularization (MNV) (6, 8). Approximately 10-15% of all AMD patients develop neovascular AMD (nAMD) (9, 10), which includes type 1 macular NV (occult choroidal NV), type 2MNV (classic choroidal NV), and type 3 macular NV (11, 12).

Vascular Endothelial Growth Factor (VEGF) signaling plays a key role in vascular development and stimulation of ocular angiogenesis (13, 14). For this reason, anti-VEGF drugs, which include ranibizumab, aflibercept, bevacizumab, brolucizumab, and the recently FDA-approved faricimab (15), were established as first-line therapy for the management of diabetic macular edema (16), PDR (17, 18) and nAMD (19, 20). Nevertheless, anti-VEGF therapy requires frequent and costly intravitreal injections and has been associated with local side effects (e.g., endophthalmitis, cataracts, retinal detachment, vitreous hemorrhage, and increased ocular pressure) (1, 21). Furthermore, some patients exhibit only a moderate to poor response after continued intensive anti-VEGF treatment (22, 23). Although several therapeutic strategies have shown significant potential in preclinical studies and clinical trials (24–26), therapies for dry AMD are still commercially unavailable and its management relies on regular follow-up evaluation, the prevention of risk factors, and increased intake of vitamins and antioxidants (8, 24). Therefore, a better knowledge of the pathological mechanisms underlying the disease onset or progression could be helpful to explore effective therapeutic alternatives for the management of these proliferative eye diseases.

So far, the characterization of the proteome of the vitreous humor in DR/PDR has contributed extensively to the identification of target pathways and candidate biomarkers for its diagnosis and treatment (27–30). However, the validation of these potential biomarkers in a larger number of samples, essential for assessing their relevance in clinical practice, is frequently unaccomplished (31). On the other hand, few studies have focused on the characterization of vitreous proteomics in AMD (32–34). To elucidate the underlying pathological mechanisms, we have applied a label-free quantitative (LFQ) proteomics approach for the understanding of the vitreous proteome in PDR and AMD compared to idiopathic epiretinal membranes (ERM) samples. A scheduled multiple reaction monitoring (MRM) method was designed for potential biomarker verification in a larger cohort of human vitreous samples.

## Materials and methods

### Collection of vitreous samples by pars plana vitrectomy

Vitreous samples were collected at the Ophthalmology Service of Leiria-Pombal Hospital, Portugal, as previously described (35), according to a protocol approved by the hospital ethics committee (Code: CHL-15481). All patients included in this study gave their informed consent, which adhered to the tenets of the Declaration of Helsinki. Vitreous samples were collected in sterile cryogenic vials at the beginning of pars plana vitrectomy by aspiration into a 2 mL syringe attached to the vitreous cutter. Upon collection, vitreous samples were placed immediately on ice and frozen at -80°C until further analysis. The medical history of the patients was assessed to confirm the diagnosis, baseline characteristics, and associated diseases. Demographic characteristics, including age and gender, and the description of corresponding vitreous samples are summarized in **Table 1** (more details in **Supplementary Table 1**). Samples from patients subjected to intraocular surgeries or intravitreal drug treatments in the previous 3 months were excluded from the study. Most patients underwent surgery for ERM removal due to the marked decrease in visual acuity. For label-free proteomic analysis, 12 patients (7 women and 5 men) diagnosed with PDR (n=4), dry AMD (n=4), and ERM (n=4) were selected. Older patients or with other serious illnesses associated (e.g., neoplasia) were removed from the study. For MRM, a larger cohort (n=65) was used, including some of the samples previously analyzed in the LFQ experiment. MRM experiments were performed on vitreous samples from patients with ERM (n=21), DR/PDR (n=20), AMD (n=11), and rhegmatogenous retinal detachment (RRD) with and without proliferative vitreoretinopathy (PVR) (n=13). Finally, 27 patients were selected for Western Blot (WB) analysis, including patients with ERM (n=5), PDR (n=9), AMD (n=6), and RRD (n=7), from which 3 patients have PVR.

**Table 1:** Demographic characteristics of patients involved in the study and description of corresponding vitreous samples collected *via* pars plana vitrectomy.

		ERM^1^ (n=25)	DR/PDR^1^ (n=21)	AMD^1^ (n=12)	RRD/PVR^1^ (n=14)
**Demographic characteristics**	Gender^2^	F=6 M=19	F=7 M=14	F=7 M=5	F=5 M=9
	Age (Mean ± SD)	73± 15	60 ± 16	78 ± 6	70 ± 13
	Age (range)	9-88	22-79	70-92	41-94
	Eye Submitted to PPV^3^	LE=10 RE=15	LE=10 RE=11	LE=6 RE=6	LE=8 RE=6
**Characterization of vitreous samples**	Protein concentration (µg/µl, MD ± SD)	0.99 ± 0.92	2.15 ± 2.10	1.12 ± 1.05	1.32 ± 1.46
**Experiments**	Label-free quantitation	n=4	n=4	n=4	____
	Verification by MRM	n=21	n=20	n=11	n=13
	Western blot analyses	n=5	n=9	n=6	n=7

^1^ERM, Epiretinal membranes; DR/PDR, Diabetic retinoaphy/Proliferative diabetic retinopathy; AMD, Age-related macular degeneration; RRD/PVR, Rhegmatogenous retinal detachment/Proliferative vitreoretinopathy. ^2^ F, Female; M, Male. ^3^ PPV, Pars plana vitrectomy; RE, right eye; LE, left eye.

### Preparation of vitreous samples

Vitreous samples were centrifuged at 18400 x g for 15 min at 4°C and supernatant protein concentration was determined using Pierce 660 nm protein assay (Thermo Fisher Scientific, Massachusetts, USA; RRID : SCR_008452), according to manufacturer’s instructions. For the removal of high-abundant proteins, High Select™ Top14 Abundant Protein Depletion Mini Spin Columns (Thermo Fischer Scientific, Massachusetts, USA; RRID : SCR_008452) were used. Briefly, 300 µl of sample (400 µg of protein) was homogeneously mixed with the resin and incubated for 10 min at room temperature. Depleted vitreous samples were recovered by centrifugation at 1000 x g for 2 min and concentrated using Nanosep^®^ Centrifugal Devices 10K (Pall, Madrid, Spain). Then, samples were solubilized with loading sample buffer, denaturized at 60 °C for 10 min, loaded, and concentrated in a 12% SDS-PAGE gel. After Quick Coomassie staining, protein bands were manually excised, cut into cubes (1 mm2), and placed in 96-well plates. In-gel tryptic digestion was performed automatically in a Proteineer DP robot (Bruker Daltonics, Bremen, Germany), as previously described (36). Tryptic peptides were extracted by adding 1% formic acid in 50% acetonitrile, collected from wells, dried by speed-vacuum centrifugation, and frozen at -20°C until further processing.

### LC-MS/MS quantitative analysis

LC-MS/MS analyses were performed using a nanoLC Ultra 1D plus (Eksigent Technologies, AB SCIEX, Foster City, CA) coupled to a SCIEX TripleTOF 5600 Mass Spectrometer System (RRID : SCR_018053) *via* a Nanospray III source. Tryptic peptides were solubilized using solvent A (2% acetonitrile [ACN] in water, 0.1% FA) and the concentration was determined using Thermo Fisher Qubit fluorimeter (RRID : SCR_018095), according to manufacturer’s instructions. Tryptic peptides (1 µg) were loaded on a C18 Acclaim PepMap™ 100 trapping column (Thermo Scientific, 100 µm I.D. × 2 cm, 5 µm particle diameter, 100 Å) using solvent A at 2 µL/min and, after desalting, switched online with an Acquity UPLC^®^ M-Class Peptide BEH C18 analytical Column (Waters, 75 µm × 15 cm, 1.7 µm, 130 Å). Peptides were fractionated at a flow rate of 250 nL/min in a 250 min gradient with increasing concentrations of ACN (2% to 90%). TripleTOF 5600 system was operated in positive ion mode as follows: ion spray voltage 2300 V, curtain gas (CUR) 35, interface heater temperature (IHT) 150°C, ion source gas 1 (GS1) of 25, and declustering potential (DP) of 100 V. Data were acquired in information-dependent acquisition (IDA) mode with Analyst^®^TF 1.7 Software (SCIEX, USA; RRID : SCR_015785). IDA parameters were: survey scan in the mass range of 350–1250 m/z, accumulation time 250 ms, followed by MS2 spectrum accumulation for 100 ms (100–1800 m/z) in a cycle of 4.04 sec. MS/MS fragmentation criteria were: ions in the 350-1250 m/z range with a charge state of 2–5 and an abundance threshold greater than 90 counts. Dynamic exclusion was set to 15s. IDA rolling collision energy (CE) parameter script was used to control the CE.

### Bioinformatics and statistical analysis

MaxQuant 1.6.5.0 (RRID : SCR_014485) was used to generate peak lists from raw files, peptide and protein identification after database search, and for LFQ intensity-based absolute quantification (iBAQ). Andromeda search engine was used to search the acquired MS/MS spectra against the UniProtKB Homo sapiens database (20418 reviewed protein sequences). Search parameters were set as follows: carbamidomethyl (C) as a fixed modification, oxidation (M), acetyl (Protein N-term), Gln→pyro-Glu and, Glu→pyro-Glu as variable modifications, trypsin/P as protease allowing up to 2 missed cleavages. Precursor mass tolerances were set at 20 ppm and the fragment mass tolerance at 0.01 Da. Proteins identified only with modified peptides (“only by site”), reversed sequences, and potential contaminants were removed. For LFQ, multiplicity was set at 1, LFQ min ratio counts at 2, and the options “iBAQ” and “match between runs” (time window of 0.7 min and alignment of 20 min) were selected. False discovery rate (FDR) of peptides and proteins was set at 1%. Additionally, mgf. files were generated using PeakView^®^ Software (AB SCIEX, RRID : SCR_015786) and searched using Mascot v.2.2.04 (RRID : SCR_014322) against the homo sapiens UniProtKB reviewed database, as described above. Search parameters were identical to those previously described but peptide mass tolerance and MS/MS fragment tolerance were set to 25 ppm and 0.05 Da, respectively. FDR of ≤1% at the peptide level was assessed by applying a target-Decoy approach. Decoy sequence database contained reversed shuffled peptide sequences (37).

The protein normalized intensity lists of the 12 vitreous samples from PDR (n=4), nAMD (n=4), and ERM (n=4) groups were processed using Perseus 1.6.10.0 (RRID : SCR_015753). The normalized intensity was calculated by dividing the intensity of each protein by the sum of the intensity of all proteins detected in that sample and multiplying it by the median of the sum of the intensity of all proteins detected in vitreous samples. Depleted proteins, potential contaminants, reversed and proteins only identified by site were removed. Data were logarithmized (Log2), filtered by valid values (min 70% of valid values), and missing values were imputed with random numbers from a normal distribution (width=0.3, shift=1.8). Multi-scatter plots and histograms were applied to evaluate data quality. *Post-hoc* tests, hierarchical clustering, principal component analysis (PCA), and two-sample t-tests were performed for differentiating the three groups in terms of protein expression based on intensity differences. A permutation-based method was used to correct for multiple hypothesis testing with the number of randomizations set to 250 and an FDR<5%. Differentially expressed proteins were analyzed using DAVID (RRID : SCR_001881) (38), and ClueGO 2.5.7 (RRID : SCR_005748) (39) for functional enrichment based on gene ontology (GO) analyses and pathways/reactions (KEGG (RRID : SCR_012773) and Reactome (RRID : SCR_003485)). Protein-protein association networks were assessed using the online tool STRING 11 (RRID : SCR_005223), with high confidence (0.75), based on the molecular action (40).

### Verification by multiple reaction monitoring

Proteotypic peptide transitions for MRM-based targeted proteomics analysis were selected using Skyline v. 19.1.0.193 (RRID : SCR_014080). MRM assays were performed in an Eksigent nanoLC Ultra 1D plus system (AB SCIEX, Foster City, CA) coupled to a SCIEX 5500 QTRAP 5500 Mass Spectrometer (RRID : SCR_020517)via a Nanospray III source. A scheduled method was designed for the relative quantitation of 35 proteins, using 2-3 proteotypic peptides per protein and 3-4 transitions per peptide (332 transitions in total). The Homo sapiens UniProtKB reviewed database was used as background proteome. The selected enzyme was trypsin/P [KR |-] and peptide parameters were set to: a length range of 8 to 25 amino acids, 2+ and 3+ charged, no missed cleavages, and potentially modified residues such as methionine (Met, M) and cysteine (Cys, C). When possible, peptides were selected to cover distinct regions of the protein sequence. As described above, 10 µg of non-depleted vitreous samples were loaded on an SDS-PAGE gel and in-gel digested. Peptide concentration was determined using Thermo Fisher Qubit fluorimeter (RRID : SCR_018095), according to manufacturer’s instructions, and 1 µg of tryptic peptides was loaded onto a C18 Acclaim PepMapTM 100 column (Thermo Scientific, 300 µm I.D. × 5 cm, 5 µm particle diameter, 100 Å) using solvent A (2%B ACN, 0.1% formic acid in water) at 2 µL/min. After desalting, the trap column was switched online with a C18 BioSphere column (Nano-separations, 75 µm I.D. × 15 cm, 3 µm particle diameter, 120 Å) and peptides were fractionated in a 30 min gradient (4 to 90% of 100% ACN, 0.1% formic acid) at 300 nL/min, followed by 15 min of equilibration to initial conditions. The 5500 QTRAP system was operated in positive polarity and MRM scan mode, with an ion spray voltage of 2800 V, IHT of 150 °C, CUR of 20, GS1 of 25, medium collision gas, and DP of 80 V. Scheduled mode was enabled and detection window set at 300 sec. Collision energy and expected retention time for each transition were defined in Skyline. Beta-galactosidase standards and a pool of vitreous samples were injected alternately with the vitreous samples to monitor oscillations in the MS signal and in the retention time. Raw MS data were imported into Skyline and the automatically selected transition peaks were manually revised considering the retention time and the intensity distribution of the selected transitions. The total area of each protein was calculated by summing the area of the respective peptides (calculated as the sum of all peptide transitions). To correct the fluctuations in MS signal over time, the calculated total area of each protein was normalized by dividing it by the total area of digested beta-galactosidase (injected between each batch) and multiplying by the median. Statistical analysis by one-way ANOVA (Tukey’s HSD) and *post-hoc* tests and multivariate statistical analyses were performed using Metaboanalyst v5.0 (RRID : SCR_015539) (41). Partial least squares discriminant analysis (PLS-DA) was used to build a predictive model to define a panel of discriminatory biomarkers of vitreoretinal diseases. The predictive ability (Q2), R-Squared (R2), and accuracy of the model were calculated *via* cross-validation to define the optimal number of components for classification. Classical univariate and multivariate exploratory receiver operating characteristic (ROC) analyses were performed to evaluate the diagnostic potential of discriminatory proteins between the disease groups. ROC curves were generated in multivariate exploratory analyses by Monte-Carlo cross-validation using balanced sub-sampling, in which two-thirds of the samples are used to evaluate the feature importance. For model building, PLS-DA was defined as classification method and PLS-DA built-in as the feature ranking method, while the number of latent variables was defined to 2.

### Western blotting assays

For western blot analysis, equal amounts of proteins (15 µg) were separated by SDS-PAGE and transferred to a PVDF membrane using the Trans-Blot Turbo™ Transfer System (Bio-Rad Laboratories, Hercules, CA, USA). After blocking with 5% of powdered milk in 0.1% Tween-20, membranes were incubated overnight at 4°C with distinct antibodies in 5% of BSA. These included 1:3000-diluted polyclonal rabbit anti-human chromogranin-A (CHGA) antibody (Agilent Cat# A0430, RRID : AB_2847855), 1:500-diluted polyclonal rabbit anti-tissue inhibitor of metalloproteinase inhibitor 2 (TIMP2) antibody (Abcam Cat# ab74216, RRID : AB_1271228), 1:1000-diluted monoclonal mouse anti-β-Amyloid (APP) antibody (Sigma-Aldrich Cat# A5213, RRID : AB_476742), and 1:500-diluted monoclonal mouse anti-cystatin C (CYTC) antibody (sc-515732; Santa Cruz Biotechnology). Thereafter, PVDF membranes were incubated with a 1:10000 dilution of anti-mouse IgG (Fab specific)–Peroxidase antibody (Sigma-Aldrich Cat# A3682, RRID : AB_258100) or anti-Rabbit IgG (whole molecule)–Peroxidase antibody (Sigma-Aldrich Cat# A0545, RRID : AB_257896). Protein bands were visualized using the Clarity Western ECL Substrate (Biorad, Hercules, CA, USA). Band detection and relative quantification were performed using Image lab 5.0 software (Biorad, Hercules, CA, USA, RRID : SCR_014210). Statistical analyses (Kruskal-Wallis tests, q-value<0.05) were performed using GraphPad Prism (RRID : SCR_002798).

## Results

### Vitreous proteome in diabetic retinopathy and age-related macular degeneration

For the discovery phase, LFQ quantitative proteomics was applied to analyze the proteome of vitreous collected from patients with DR (n=4), dry AMD (n=4), and ERM (n=4). Using two different strategies for protein database search, a total of 680 proteins were identified, of which 586 proteins were identified by MASCOT and 580 proteins by MaxQuant (corresponding to 474 protein groups) (**Supplementary Table 2**). The mass spectrometry proteomics data have been deposited to the ProteomeXchange Consortium *via* the PRIDE (42) partner repository with the dataset identifier PXD038285. An average of 366 ± 31 protein groups was identified in control ERM vitreous, 361 ± 46 protein groups in dry AMD, and 310 ± 14 protein groups in PDR. A total number of 195 protein groups were detected in all the samples. Multiple scatter plots (**Supplementary Figure 1**) were applied to assess data reproducibility and correlation within and between disease groups and showed high data reproducibility. The best correlation values between replicates and within groups was found for PDR samples (average 0.89 ± 0.02), but samples from dry AMD and ERM groups also showed good correlation values within groups (average 0.87 ± 0.05). Sample correlation was higher within than between groups, except for the dry AMD group. One of the samples collected from a patient with dry AMD (VH 219) showed a poorer correlation (< 0.8) with other dry AMD samples and a higher correlation with samples from the PDR group (> 0.87). For this reason, this sample was removed from quantitative analysis, improving within-group Pearson correlation values from an average of 0.84 ± 0.06 to 0.90 ± 0.01 (**Supplementary Figure 1**).

**Figure 1:**
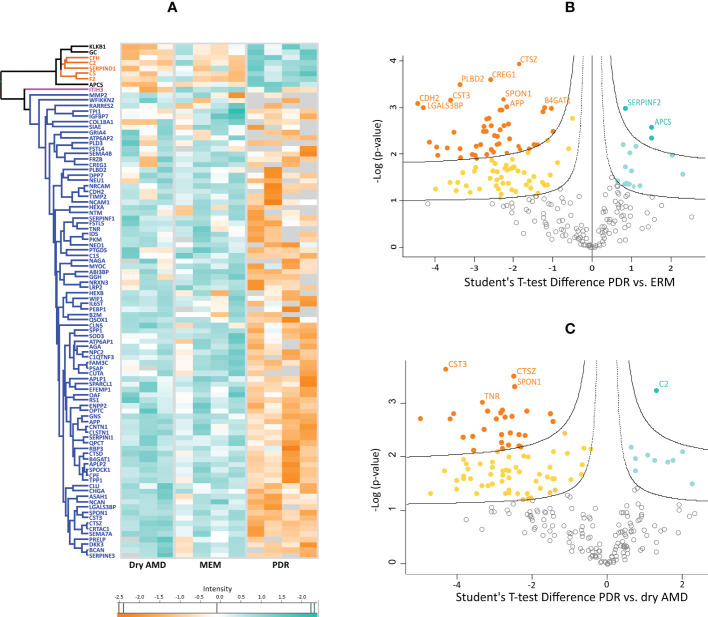
**(A)** Heatmap representing the intensities normalized to log base 2 and analyzed by hierarchical clustering of 96 proteins found differentially expressed among the three disease groups in posthoc tests. Hawaii plots display the proteins found up-regulated (blue-green) and down-regulated (orange) in proliferative diabetic retinopathy (PDR) compared to patients with **(B)** epiretinal membranes (ERM) and with **(C)** dry age-related macular degeneration (dry AMD).

Subsequently, *post-hoc* tests and hierarchical clustering were performed to differentiate the three groups in terms of protein expression based on intensity differences. *Post-hoc* tests revealed that 96 proteins are differentially expressed among the three disease groups. Specifically, 83 and 79 proteins differed between PDR and ERM or between PDR and dry AMD groups, respectively (**Supplementary Table 3**). Hierarchical clustering analysis of these 96 proteins is represented in a heatmap based on their intensities normalized to log base 2 (**Figure 1A**). **Figure 1A** reveals that most of these proteins are downregulated in PDR compared to ERM and dry AMD groups (blue cluster), except for a small cluster (orange cluster) composed mainly of complement (C5, C2, CFH) and coagulation factors such as prothrombin (THRB), among other proteins. Only inter-alpha-trypsin inhibitor heavy chain H3 (ITIH3) differentiates the dry AMD from ERM control samples in hierarchical clustering, as represented in a pink cluster (**Figure 1A**). These proteins were compared with those found differentially expressed in vitreous from DR and AMD in previous proteomics studies (**Supplementary Table 3**). Furthermore, multiple t-tests with an FDR cutoff of 5% were performed to identify differentially expressed proteins in PDR versus dry AMD and PDR versus ERM (**Figures 1B, C** and **Supplementary Table 3**). We found 118 significantly regulated proteins (17 up- and 101 down-) and 95 proteins (10 up- and 85 down-) in PDR relative to the ERM and dry AMD groups, respectively. Most of the proteins (5 up- and 76 down-regulated proteins) differentiated between PDR and either of the two other diseases, but 14 and 37 proteins were unique to the comparison with the dry AMD and ERM groups, respectively. Fetuin-B, keratin, type II cytoskeletal 2 epidermal, and serum amyloid P-component showed the highest levels of expression in PDR (FDR<0.001), while the more significant down-regulated proteins were protein CREG1, neural cadherin (CADH2), galectin-3-binding protein (LGALS3BP), Putative phospholipase B-like 2, and CYTC. LGALS3BP and CYTC were also significantly downregulated in PDR versus dry AMD, as well as cathepsin Z (CATZ), spondin-1 (SPON1), and tenascin-R (TNR). Complement C2 (C2) showed the most statistically significant change in the PDR group compared to dry AMD with an FDR lower than 0.001 (**Figure 1C**). Other complement factors (e.g., CFB, C8B), acute-phase response proteins (e.g., alpha-2-antiplasmin), and proteins related to lysosomal degradation (e.g., alpha-N-acetylgalactosaminidase, Prosaposin, Cathepsin L1) and ECM organization (metalloproteinase inhibitor 1 [TIMP1]) showed differential expression only in PDR compared to ERM. Although some proteins such as ProSAAS, neurosecretory protein VGF, or phosphoglycerate mutase 1 were exclusively detected in dry AMD samples, no differential proteins were found compared to ERM controls with an FDR<5%.

### Functional enrichment of differentially expressed proteins

**Figure 2:**
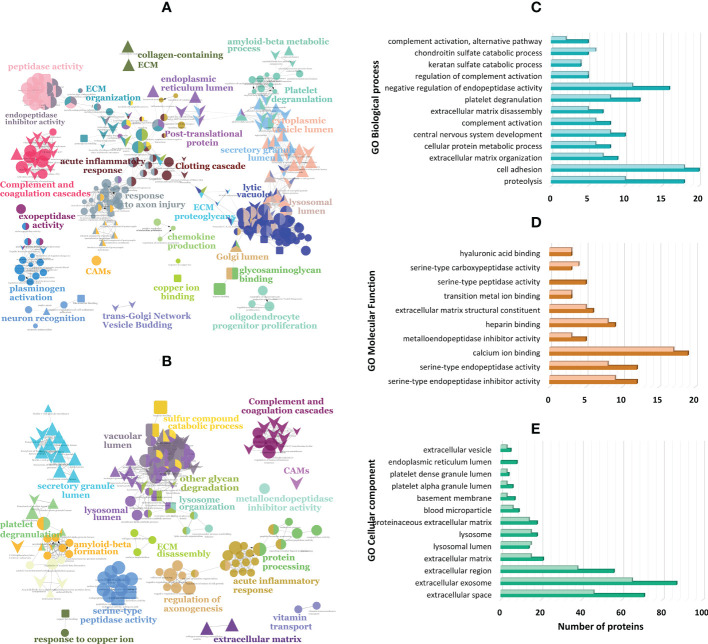
Functionally grouped network of enriched categories generated in ClueGO (Cytoscape app) for the proteins found differentially expressed in proliferative diabetic retinopathy (PDR) compared to patients with **(A)** epiretinal membranes (ERM) and with **(B)** dry age-related macular degeneration (dry AMD). These proteins were also classified according to gene ontology (GO) terms using DAVID Bioinformatics tool and ClueGo for **(C)** biological process, **(D)** molecular function, and **(E)** cellular component with the darkest colors representing proteins differentially expressed in PDR versus ERM, and lightest colors representing proteins differentially expressed in PDR versus dry AMD.

To gain insights into the biological roles and pathways of the differentially expressed proteins, these were analyzed using bioinformatics tools such as DAVID, ClueGo (Cytoscape app), and STRING. Functional enrichment indicates that proteins underexpressed in PDR are highly correlated and share common biological processes/pathways such as extracellular matrix (ECM) disassembly and organization, platelet degranulation, lysosomal degradation, cell adhesion, and central nervous system development (e.g., regulation of axon regeneration) (**Figures 2A, B** and **Supplementary Table 4**). Some of these underexpressed proteins, including beta-hexosaminidase (HEXA, HEXB) and N-acetylglucosamine-6-sulfatase (GNS), are involved in chondroitin sulfate and keratan sulfate catabolic processes (**Figure 2C**). Furthermore, some of the proteins involved in these processes also participate in cell adhesion and ECM organization (e.g, brevican core protein [BCAN] and neurocan core protein [NCAN]) or are themselves ECM components such as prolargin. In turn, proteins related to acute-phase responses and fibrin clot formation are only found upregulated in PDR compared to ERM, whereas complement and coagulation proteins are up-regulated in PDR in both comparisons. According to GO classification for molecular function (**Figure 2D**), both up- and downregulated proteins in PDR have serine-type endopeptidase activity (7.0- and 6.0-fold enrichment), serine-type endopeptidase (18.5- and 17.6-fold) and metalloendopeptidase inhibitor activities (46.7- and 35.6-fold), or binding function (e.g., heparin and calcium). On the other hand, down-regulated proteins have serine-type carboxypeptidase activity (32.0- and 54.2-fold) and/or binding functions or are ECM structural constituents (13.4- and 14.2-fold). According to GO classification for cellular components (**Figure 2E**), most of the differentially expressed proteins are localized extracellularly and, notoriously, many of them are associated with extracellular exosomes (87 proteins). A significant part of downregulated proteins in PDR compared to ERM and dry AMD are in the basement membrane (13.7- and 7.3-fold enrichment), the lysosomal lumen (25.4- and 33.9-fold enrichment), and secretory vesicles, such as platelet dense granule (44.1 and 41.1-fold enrichment), whereas overexpressed proteins are mainly blood microparticles. Many of the proteins found underexpressed in PDR compared to ERM and dry AMD are related to ECM with fold enrichment of 11.0 and 9.7, respectively. Although less significant, many underregulated proteins are localized in the neuronal cell body, axons, node of Ranvier, perineuronal nets, and postsynaptic membranes.

Differentially expressed proteins were collated using the DAVID bioinformatics tool (**Supplementary Table 4**). Proteins involved in pathways such as complement and coagulation cascades, lysosomal degradation, ECM organization, and regulation of inflammatory response, were found associated with type 2 diabetes and macular edema, whereas the pigment epithelium-derived factor (PEDF) and serine protease HTRA1 were found specifically related to DR. These two proteins, as well as complement components (e.g., CFH, C2), regulators of complement cascades (clusterin (CLU)) and amyloidosis proteins (amyloid-beta precursor protein [APP], CYTC3) are also associated to macular degeneration and pathological processes, such as choroidal neovascularization, geographic atrophy, and retinal drusen.

**Figure 3:**
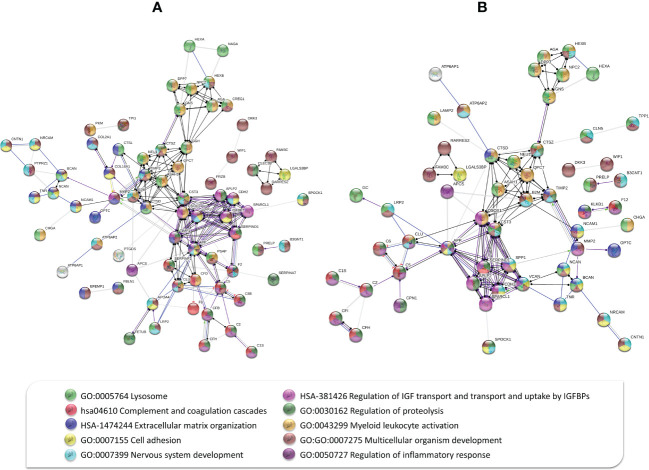
Protein-protein interaction network between **(A)** 118 differentially expressed proteins in proliferative diabetic retinopathy (PDR) versus epiretinal membranes (ERM), and **(B)** 95 differentially expressed proteins in PDR versus dry age-related macular degeneration based on molecular action with high confidence (0.70). Some biological processes/pathways stand out in both interaction networks, including complement and coagulation cascades, lysosomal degradation, cell adhesion, extracellular matrix organization, multicellular organism, and nervous system development, among others, as represented by colored nodes.

In addition, STRING was used to generate high-confidence (0.70) protein-protein interaction networks between the 118 and 95 differentially expressed proteins in PDR versus ERM (**Figure 3A**) and PDR versus dry AMD, respectively (**Figure 3B**). Some biological processes/pathways stand out in both analyses, including multicellular organism and nervous system development, myeloid leukocyte activation, regulation of proteolysis, and cell adhesion, as well as proteins associated with lysosomes. These data reinforce that ECM organization, complement and coagulation cascades, and inflammatory responses are relevant in these diseases. Specific pathways/terms such as regulation of insulin-like growth factor (IGFs) transport and uptake by insulin-like growth factor binding proteins (IGFBPs), amyloidosis, neurodegeneration, metabolism of angiotensinogen to angiotensin, post-translational modifications (e.g., phosphorylation), and regulation of Wnt signaling and MAPK cascades were also found associated to differentially expressed proteins. Remarkably, several proteins, including APP, CLU, CYTC, CATZ, osteopontin (OSTP), TIMP2, and lecticans (e.g., NCAN and BCAN), play key roles in multiple pathways, as seen in **Figure 3**.

### Selection of potential biomarkers for verification by multiple reaction monitoring

Several potential biomarkers were selected for further verification by targeted proteomics (MRM). Selection was performed according to their statistically significant differential expression levels found in the discovery phase (LFQ experiment), their interaction with other proteins as described in STRING interaction network, and the pathways in which they are involved. Among the selected biomarkers there are proteins involved in complement and coagulation cascades, amyloidosis, ECM organization, cell adhesion, and lysosomal enzymes, among others (**Supplementary Table 5.1**). Selection criteria considered the number of unique (specific) peptides detected for each protein and the signal-noise ratio in the fragmentation spectrum to increase the probability to detect the proteins by MRM in non-depleted vitreous samples. Some of the biomarkers reported in previously published proteomics studies, such as alpha-1-antitrypsin (A1AT), were also considered. A1AT, which was depleted in the discovery phase, is an acute-phase protein highly abundant in vitreous that was selected as a potential biomarker according to previous studies performed in AMD (32), RD/PVR (43–45), and DR/PDR (43, 46–50). Some of the biomarkers selected from proteins found differentially expressed here were reported in our previous study (51), including retinoschisin (XLRS1) and LGALS3BP. Both were found upregulated in rhegmatogenous retinal detachment (RRD) compared to ERM, while coagulation factor IX (F9) and complement components C8 chain (C8) and C2 were found down-regulated. Alpha-1-antichymotrypsin (AACT) and complement component C9 (C9), non-reported as differential in the present study, were also considered for verification. Therefore, a group of patients with RRD, without and with PVR, were included in this verification. MRM experiments were performed on vitreous samples from patients with ERM (n=21), DR/PDR (n=20), AMD (n=11), and RRD/PVR (n=13). The final scheduled MRM method including the list of potential biomarkers and the corresponding peptides and transitions monitored, as well as other parameters, is detailed in the **Supplementary Table 5.1**.

Of the 35 proteins analyzed, MRM results for TIMP2 and CHGA were not considered for quantitation due to the poor-quality quantitative data. CHGA was undetected in many samples, whereas in the case of TIMP2 only a peptide could be detected with few transitions. However, these proteins were analyzed by WB analysis (**Supplementary Figure 3**). CHGA was found downregulated by LFQ in patients with PDR compared to dry AMD and ERM, but this result could not be confirmed by WB analysis. In opposition to the LFQ results, MRM analysis showed that TIMP2 levels are increased in PDR compared to ERM and AMD groups. Additionally, TIMP2 levels are significantly lower in RRD/PVR group, and it was not even detected in two samples (HV 500 and HV 785).

### Evaluation of discriminatory biomarkers of vitreoretinal diseases

**Figure 4:**
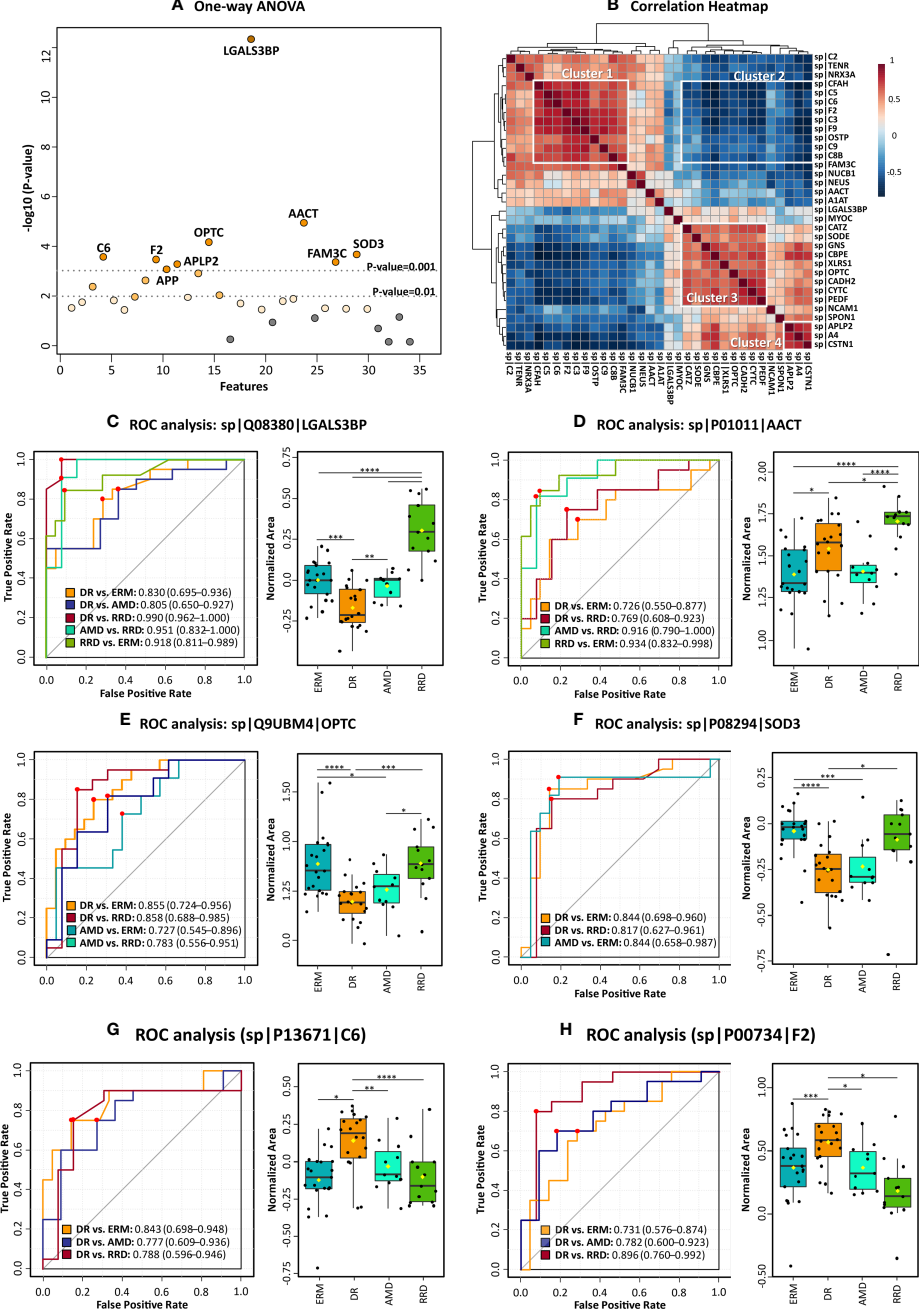
Statistical analysis of candidate vitreous biomarkers analyzed by multiple reaction monitoring was performed by MetaboAnalyst5.0 **(A)** One-way ANOVA plot with the more statistically relevant biomarkers highlighted with different orange grades (stronger colors represent more significant p-values). **(B)** Heatmap showing the correlation between different candidate biomarkers. **(C–H)** Results of univariate biomarker analysis of the top six candidate biomarkers in one-way ANOVA analysis, including the ROC curves for statistically significant comparisons between groups and respective box plots. Data is presented in the box plots as median ± SD and the statistical analysis was performed by two-sample t-tests, with the symbol asterisk determining the statistical significance. *p-value<0.05, ** p-value<0.01, ***q-value<0.001, and ****q-value≤ 0.0001.

One-way ANOVA, *post-hoc* tests, and classical univariate ROC curve analyses were performed in MetaboAnalyst to evaluate the potential of the candidate biomarkers to discriminate between the different vitreoretinal diseases understudy (**Supplementary Table 5**, **Figure 4A**). One-way ANOVA and *post-hoc* tests showed that 26 out of the 35 analyzed proteins were verified as differential between the different groups. ROC analysis of PEDF, A1AT, and NUCB1 showed that these biomarkers also have the power to discriminate between groups accordingly, but they show more modest area under the curve (AUC) values compared to other biomarkers (0.7<AUC<0.8, p-value<0.05). Furthermore, correlation analysis showed that there is a strong correlation between the levels of these candidate biomarkers, standing out of 4 correlation clusters (**Supplementary Table 5.5**; **Figure 4B**).

Among all quantified proteins, LGALS3BP, AACT, and OPTC showed the highest potential to differentiate between groups with AUC>0.85 (ANOVA p-value<0.0001), as seen in **Figures 4C–E**. The high levels of LGALS3BP found in RRD groups by MRM analysis confirms our previous data (51). Therefore, this biomarker candidate could distinguish proficiently RRD patients from the other disease groups (0.8>AUC>0.99, p-value< 5,00E-03). The LGALS3BP downregulation in DR compared to ERM and AMD has also been confirmed, allowing this candidate biomarker to distinguish this disease from ERM (AUC=0.83, p-value=1.02E-04) and AMD (AUC=0. 80, p-value=4.61E-03). AACT was found to be upregulated in RRD compared to ERM, in contrast with our previous iTRAQ results (51). The highest levels of AACT were found in the RRD group, discriminating this group from the others with high efficiency, especially from ERM (AUC=0.93, p-value=6.94E-06) and AMD (AUC=0.92, p-value=4.55E-05). The downregulation of OPTC in DR compared to both AMD and ERM was also verified. OPTC allowed to efficiently differentiate DR (AUC≥0.85, p-value ≤ 1.23E-04) and less efficiently AMD (AUC≥0.73, p-value ≤ 0.05) from ERM/RRD. Furthermore, the correlation heatmap (**Figure 4B**) shows that OPTC levels correlate inversely with coagulation and complement components (cluster 2). However, OPTC correlated directly with other proteins that were also verified as downregulated in DR (cluster 3). The last ones include carboxypeptidase E (CBPE), CYTC, and the extracellular superoxide dismutase [Cu-Zn] (SOD3), as well as vitreous antiangiogenic factors (PEDF), lysosomal enzymes (GNS), and cell adhesion factors (CADH2, XLRS1, and CSTN1). All these proteins were capable of differentiating DR from ERMs (AUC≥0.71, p-value ≤ 0.05), whereas PEDF, CYTC, XLRS1, and SOD3 also can discriminate patients with DR from RRD (AUC≥0.75, p-value ≤ 2.15E-02). Indeed, SOD3 showed to be one of the more efficient biomarkers in distinguishing DR from ERM/RRD groups (0.82≤AUC ≤ 0.84; p-value ≤ 1.41E-02) and one of the few biomarkers to differentiate AMD from ERM (AUC=0.84, p-value=9.33E-04).

Also related to cluster 3, the downregulation of several adhesion molecules in PDR compared to ERM/AMD was confirmed by MRM, except for neurexin-3 (NRX3A), whose PDR levels were higher than in ERM (**Supplementary Table 5.2**). NRX3A, along with SOD3 and OPTC, is unique in its ability to differentiate between AMD and ERM groups (AUC=0.75, p-value=1.51E-02). Several adhesion molecules are capable to discriminate between DR and ERM (**Supplementary Table 5.4**) but CDH2, calsyntenin-1 (CSTN1), and LGALS3BP showed better efficacy with AUCs higher than 0.76 (p-value<1.00E-02). In turn, CSTN1 and LGALS3BP showed a great potential to differentiate between DR and AMD with AUC of 0.81 (p-value=1.16E-03) and 0.80 (p-value=1.57E-03), respectively. In turn, the lowest SPON1 levels were detected in the RRD group, confirming this protein as a good discriminatory biomarker when compared to AMD (AUC=0.81, p-value=6.46E-03) and ERM groups (AUC=0.80, p-value=7.65E-03), but not from DR. MRM analysis could not confirm the downregulation of OSTP in DR, but distinguished RRD from DR very efficiently (AUC=0.87, p-value=1.63E-04).

The upregulation of several complement and coagulation components was confirmed in DR versus ERM and AMD, which allows differentiate DR from the other disease groups with high sensitivity and specificity (AUC≥0.69, p-value<0.05). Furthermore, the levels of these components correlated positively (cluster 1, **Figure 4B**). However, correlation was negative (correlation value<-0.7, p-value<1.00E-04) with CSTN1, CBPE, GNS, and A4 levels (cluster 2, **Figure 4B**). In fact, these factors can only distinguish between RD and other pathologies. C6, CFH, and C5 showed more efficiency to differentiate between DR and ERM, with AUC≥0.83 (p-value ≤ 1.00E-03), but CFH was not capable to differentiate DR from the other groups. On the other hand, we could not confirm the previously reported downregulation of C8B, C9, and F9 in RRD (51) in comparison to ERM/AMD, but these differences are significant when compared to DR, allowing to differentiate them (AUC≥0.75, p-value<0.05). THRB discriminated very efficiently patients with DR from RRD (AUC=0.90, p-value=3.75E-05), whilst coagulation factor IX (F9) distinguished reliably patients with DR from AMD (AUC=0.87, p-value=5.64E-04) and RRD (AUC=0.80, p-value= 1,35E-03). Although the levels of most of the complement and coagulation components are very similar in these two pathologies, DR and AMD groups could be distinguished with AUC≥0.76 (p-value≤ 0.05) by several proteins, including F9, THRB, C5, and C3.

**Figure 5:**
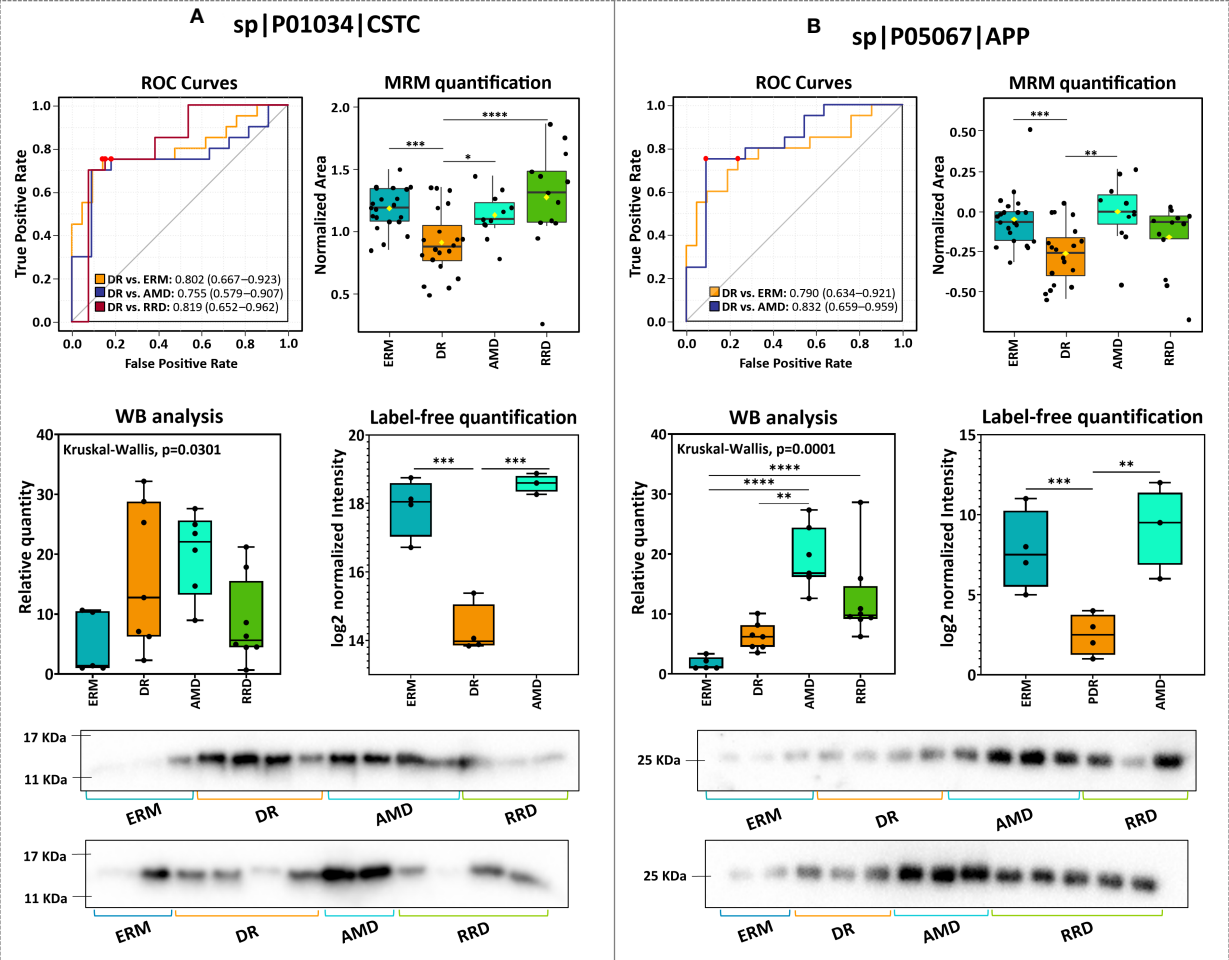
Comparison of the results of the analysis of **(A)** cystatin-C (CST3) and amyloid-beta (APP) by label-free quantitation (LFQ), multiple reaction monitoring (MRM), and western blot (WB), and respective ROC curves.

Another small cluster is related to proteins associated with amyloidosis (**Figure 4**). Downregulation of CYTC, APP, and amyloid-like protein 2 (APLP2) was confirmed by MRM (**Supplementary Table 5**, **Figure 5**). Noticeably, changes in expression levels of APP and APLP2 are quite similar, showing a high correlation value (Pearson correlation=0.80). The highest levels were found in AMD, and this difference is highly significant when compared with DR and RRD groups, where the levels are the lowest. The analysis of CYTC by MRM confirmed its statistically significant downregulation in DR samples compared to ERM and AMD groups, as well as to RRD. APP, CYTC, and APLP2 discriminate efficiently DR from ERM (AUC≥0.78, p-value<1.00E-03) and AMD (AUC≥0.75, p-value<0.05). CYTC is capable to differentiate RRD from DR (AUC=0.82, p-value=4.53E-03), whereas APLP2 differentiated this group from ERM (AUC=0.72, p-value=7.16E-03) and AMD samples (AUC=0.84, p-value=1.36E-02). APP and CYTC expression levels were also confirmed by WB analysis (**Figure 5** and **Supplementary Figures 3**), showing the downregulation of both proteins in PDR compared to AMD, but not to ERM controls. Interestingly, the APP precursor was not detected in WB analysis but two bands corresponding to APP fragments, a strong band at 25 kDa (APP fragment), and a faint band between 48 kDa and 63 kDa (**Supplementary Figure 3.1**).

**Figure 6:**
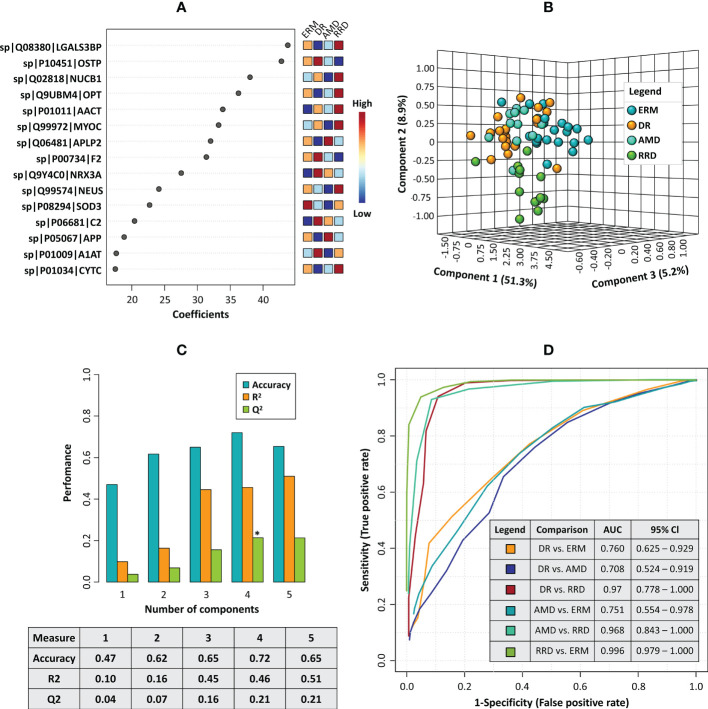
**(A)** The plot of the top fifteen candidate biomarkers according to the partial least squares discriminant analysis (PLS-DA) model. **(B)** 3D principal component analysis (PCA) plot shows the separation between disease groups based on the top fifteen candidate biomarkers panel **(C)** Barplot representing the values of predictive ability (Q2), R-Squared (R2), and accuracy calculated for the PLS-DA model in the cross-validation analysis. **(D)** Combined ROC curves showing the results of multivariate receiver operating characteristics for the panel of 15 candidate biomarkers, including the area under the curve (AUC) and the confidence intervals (95% Cl) for each comparison between different disease groups.

Considering these results, partial least squares discriminant analysis (PLS-DA) was used to build a predictive model to define a panel of discriminatory biomarkers of vitreoretinal diseases. Using this strategy, a panel of fifteen candidate biomarkers was defined (**Figure 6A**). PLS-DA model showed high predictive ability (Q2) and accuracy (**Figure 6C**). According to 3D PCA, this biomarker panel is capable to separate all vitreoretinal conditions, although this separation is more evident for RRD group (**Figure 6B**). Furthermore, multivariate Exploratory ROC analysis were performed to assess the sensitivity/specificity of this panel of biomarkers (**Figure 6D**). In all comparisons between groups, the use of all 15 proteins from the panel provides the best predictive accuracy, although this value (>90%) is better when RRD group is compared with the other (**Supplementary Figures 4**). Multivariate ROC curves showed a very good sensitivity/specificity for all comparisons but the best results were obtained for RRD group compared with ERM (AUC=0.996), DR (AUC=0.97), and AMD (AUC=0.968) (**Figure 6D**).

## Discussion

The study of the vitreous proteome has gained increasing interest to understand the pathophysiological mechanisms underlying eye diseases. Many researchers have contributed to the characterization of the human vitreous proteome in diverse pathologies, such as DR (27–30), AMD (32–34), RRD (51–55), PVR (44, 45, 56, 57), and ERM (58–62). Although the study of the vitreous proteome promises to elucidate some of the pathogenic mechanisms underlying vitreoretinal diseases, the demand for reliable vitreous biomarkers has not yet been met (63, 64). Therefore, the validation of the proposed biomarkers in a large number of samples could be decisive to assess their relevance in clinical practice (31). In this work, an LFQ-based method was applied first in the discovery phase to compare the vitreous proteome in patients affected with PDR, dry AMD, and ERM with the aim of unraveling the pathophysiological mechanisms of these diseases. This approach allowed us to identify 118 proteins differentially expressed (17 up- and 101 down-) in PDR compared to ERM patients, whereas 95 proteins (10 up- and 85 down-) were found differentially expressed in PDR compared to nAMD patients. Functional enrichment analyses suggested that proteins up-regulated in PDR are mainly associated with immune system biological processes such as complement, coagulation cascades, and acute-phase responses. On the contrary, the analysis of downregulated proteins shows that vitreous from ERM and nAMD compared to PDR patients are enriched in adhesion and neuronal proteins, lysosomal proteases, and other proteolytic enzymes, as well as in ECM components. In turn, no significant differences were observed comparing the vitreous of patients with AMD and ERM (only ITIH3 differentiates these diseases), suggesting that these diseases share common pathophysiological mechanisms. Moreover, one of the dry AMD samples correlated more strongly with PDR samples rather than with samples of its own pathological group. This suggests that, at an earlier stage, AMD pathogenesis could share common molecular mechanisms with ERM, but after progressing to a proliferative etiology, it partially resembles PDR. Our assumption is that differences found in the vitreous proteome could be considered as a source of potential biomarkers for better stratification of eye diseases. Consequently, we selected several candidate biomarkers for further verification by MRM-based targeted proteomics. From the initial list of 35 candidate biomarkers, MRM verified 26 proteins involved in diverse biological processes and with the potential to differentiate between the disease groups.

The low levels of complement and coagulation cascades in the eye are characteristic of its immune-privileged status, contributing to retinal homeostasis and integrity (65–67). Chronic activation of complement and coagulation pathways has been implicated in a variety of pathophysiological features, including increased vascular permeability (68, 69), loss and activation of choriocapillaris endothelial cells (70, 71), inflammation (71, 72), and loss of photoreceptors (66). From the first experiments using quantitative proteomics techniques, several complement components have been reported to be upregulated in DR/PDR (**Supplementary Table 3.3**) (34, 47, 48, 73–75). Although the exact role of complement and coagulation cascades in DR remains unclear, some studies have suggested that there is dysregulation and activation of the alternative pathway (76, 77). Likewise, the activation of the complement was reported in RD/PVR (44, 45, 52, 78, 79), as well as its involvement in pathological processes, such as increased vascular permeability, endothelial cell proliferation, migration, RPE atrophy, reactive gliosis, and loss of photoreceptor outer segments (45, 66). On the other hand, genetic studies strongly support the association between complement components (e.g., CO3, CFH, and CFB) and the risk for AMD (80–82). Therefore, modulation of the complement system could represent a therapeutic alternative to target ocular inflammation in AMD disease (83). However, few quantitative studies in nAMD vitreous have reported changes in complement-specific factors (34). We show here that several complement and coagulation components are significantly up-regulated in DR/PDR and AMD, reinforcing their role in these diseases. Our LFQ approach showed that complement factors such as C2 and CFH are up-regulated in PDR compared to AMD in the LFQ experiment, but this difference could not be confirmed when a larger set of samples were analysed by MRM. Schori and co-workers also reported the enrichment of complement cascade components in PDR vitreous but found reduced levels of CFH in nAMD (34). These results suggest that these complement factors increase gradually in vitreous as the disease progresses from a non-proliferative to a proliferative etiology. Indeed, higher levels of complement and coagulation factors were detected in severe forms of DR and AMD associated with neovascularization, fibrovascular proliferation, vitreous-macular traction syndrome, macular edema, and vitreous hemorrhage. In contrast, complement C1s subcomponent was found to be downregulated in PDR compared to ERM/dry AMD in LFQ, as well as lower levels of complement factor D (previously reported to be up-regulated in DR/PDR (34, 48, 74)) and complement factor I were found in PDR compared to ERM and dry AMD, respectively. Some complement and coagulation proteins had been already detected as downregulated in RRD/PVR compared to other pathologies under study as described by our group (51) and other research groups (52, 54). It has been suggested that the intravitreal levels of complement and coagulation factors increase in RRD as the disease progresses to PVR (51) due to the increased influx of plasma proteins into the retina and vitreous cavity as a result of the breakdown of the blood-retinal barrier (84, 85). Although the number of samples is insufficient to assess the statistical differences between vitreous from RRD and PVR patients, the highest levels of complement and coagulation components were found in a patient with re-detachment associated with PVR. Therefore, the increase of these proteins in vitreous is non-specific for a particular disease but could be a suitable predictor of its progression to a proliferative etiology.

However, the role of complement and coagulation cascades in vitreoretinal diseases could be more complex, as they may be involved in pathological processes shared by DR/PDR, AMD, and RRD/PVR, as recently reviewed by our group (86). In healthy neurosensory tissues, activated complement components can act as neurotrophic and anti-inflammatory factors, promoting cell survival and tissue remodeling. Nonetheless, unrestricted activation of complement cascades may cause direct damage to retinal tissue, as well as the recruitment of immune cells, thus contributing to inflammation and neurodegeneration (87). In this work, we report a significant number of adhesion molecules, nervous system development proteins, and ECM components up-regulated in dry AMD and ERM (cluster 3, **Figure 4B**), reinforcing the well-known neurodegenerative nature of these pathologies (33, 59, 61, 62), Interestingly, many of them were found to be inversely correlated with coagulation and complement components (cluster 2, **Figure 4B**). OPTC was one of the ECM components found downregulated in PDR and validated by MRM and it differentiated very efficiently DR and AMD from ERM/RRD. This glycoprotein is highly abundant in vitreous where it exerts its anti-angiogenic activity by regulating the adhesion characteristics of ECM components through its competitive binding to collagen, inhibiting endothelial cell interactions, and preventing the strong adhesions required for pro-angiogenic signalling (88). The downregulation of OPTC had already been reported in PDR in comparison to healthy and surrogate controls (50, 75, 89). Similarly, lower levels of OPTC were observed in patients with nAMD, with levels being lower in patients with more advanced degrees of CNV (33). The decrease of the levels of OPTC in vitreous, which was verified both in PDR and AMD groups, might conduce to an angiogenic environment in the eye. This hypothesis was reinforced by the downregulation of PEDF, whose levels were closely related to those of OPTC, in PDR compared to ERM and AMD, although we only confirmed this result in DR *vs* ERM. PEDF is mainly secreted by the RPE and is a potent inhibitor of angiogenesis, although it participates in other processes such as neuronal differentiation in retinoblastoma cells, inhibits retinal inflammation, and protects retinal neurons from light-induced damages, oxidative stress, and glutamate excitotoxicity (90, 91). PEDF levels in the vitreous of patients are controversial as they are not consistent across studies in PDR (47, 49, 50, 73, 89, 92–94) and nAMD (33, 95, 96). Nevertheless, the antiangiogenic and neurotrophic activities of PEDF are not only controlled by its expression levels, but also by changes in the phosphorylation levels (97, 98), which could explain these discrepancies. Another protein found positively correlated with the levels of OPTC and PEDF is SOD3, an enzyme with antioxidant activity. It was found downregulated both in the discovery phase and MRM verification in all disease groups compared to ERM controls, but these changes were more significant when we compared DR/PDR and ERM. It has been suggested that SOD3 is locally sequestered in vitreous ECM through binding to heparan sulfate proteoglycans (e.g., heparin), in areas of oxidative stress to protect retina and neighboring structures from superoxide radical-induced damages (99). Impaired redox balance in vitreous has been implicated in DR, AMD, and PVR, as reviewed recently by our group (100). Although SOD3 exerts its protective effect by removal of superoxide radicals, it has been suggested that it also promotes the survival of starving photoreceptor cells by enhancing glucose availability (101), stabilizing the retinal vasculature and reducing vessel leakage through the stabilization of hypoxia-inducible factors (102). Other proteins that belong to cluster 3 are cell adhesion factors. Our MRM results confirmed the up-regulation in AMD and ERM of proteins involved in neuronal cell adhesion (CSTN1, CDH2, SPON1), retinal cell-cell adhesion (XLRS1), and integrin-mediated cell adhesion (LGALS3BP), suggesting that these proteins are potential biomarkers to discriminate between DR and the other disease groups. In particular, LGALS3BP differentiated very efficiently all the disease groups, with the highest levels found in RRD/PVR and the lowest in PDR, as previously reported (44, 51, 75). Cell adhesion molecules participate in a wide number of biological processes in central nervous system development and retina, including neurogenesis, neuronal cell migration, and differentiation, formation and regeneration of axons, and formation of synapses and complex of glial networks synapse (103, 104). The role of adhesion molecules is supported by ECM that provides a scaffolding *via* ECM–integrin-binding for cell migration (105). Besides controlling basic cellular activities (106, 107), ECM remodeling modulates pathological features of vitreoretinal diseases like neovascularization (108, 109), inflammation (110, 111), and fibrosis (112, 113). ECM degradation mediated by metalloproteinases (MMPs) provides scaffolding areas that enable cell adhesion and migration, but also promotes changes in the bioavailability of factors sequestered in ECM, including growth factors, chemoattractant, and other signaling molecules (106, 108, 109). We found both MMP2 and TIMP2 downregulated in PDR compared to ERM and dry AMD. In turn, TIMP1 was found downregulated in PDR with respect to ERM, but not with dry AMD. In the most extreme cases, some of these molecules were not even detected in several PDR vitreous samples. However, western blot analysis could detect higher levels of TIMP2 in DR/PDR and AMD groups. It has been suggested that TIMP2 is constitutively expressed in the human retina in physiological conditions, but its expression levels change in response to a pathological stimulus (114). Zou and co-workers have reported that TIMP2 is downregulated in PDR but treatment with ranibizumab increases its expression levels, confirming its relevance as an inhibitor of angiogenesis (75). OSTP is a matricellular protein, acting both as a soluble cytokine or as an immobilized ECM compound that mediates cell migration, cell-matrix adhesion, and survival of many cell types, inflammatory responses, angiogenesis, and tissue remodeling (115, 116). OSTP was found downregulated in PDR in the discovery phase, in agreement with previous reports (34, 75, 89). However, these results were only partially confirmed by MRM, where the levels were only found downregulated in DRR/PVR group. Higher intravitreal levels of OSTP have been reported in PDR compared to RD, especially in patients with active PDR, suggesting a role of these proteins in angiogenesis (117).

Another small cluster of proteins (cluster 4, **Figure 4B**) that suggest the role of neurodegeneration in these retinal diseases are APP and related proteins (e.g., CYTC, CLSTN1, SPP1, APLP2). They form a cluster of interacting proteins that integrate multiple pathways, both in the protein-protein interaction network (**Figure 3**) and correlation heatmap (**Figure 4B**). APP is a membrane glycoprotein produced by retinal ganglion cells and the RPE that is important for neurite growth, neuronal adhesion, and axonogenesis. APP processing results in the accumulation of amyloid fragments in the eye, in particular in drusen, which have been associated with neurodegeneration in retinal diseases such as AMD and glaucoma (118–120). The increased phagocytic capacity of microglia and the expression of APP degrading enzymes have been suggested to contribute to the amyloid-beta clearance in physiological conditions. Notwithstanding, the neurotoxicity associated with the generation of APP peptides seems to be mediated by its intralysosomal accumulation through macroautophagy, and consequent lysosomal membrane permeabilization, promoting the neuroinflammation by the induction of pro-inflammatory cytokines and NLRP3 inflammasome (121–123). Furthermore, APP peptides can induce mitochondrial dysfunction, oxidative stress, the activation of the complement cascade, and changes in the vascular endothelium in the retina (120, 124). In this work, APP and APP-like proteins (e.g., APLP2) were found upregulated in AMD in comparison to DR/PDR, PDR, and RRD/PVR, and the results were verified by MRM. Several authors reported the underexpression of these proteins in PDR (34, 47, 48, 75), whereas Yu and co-workers detected them in moderate but not in severe PVR or healthy controls (44), confirming our data. Considering that APP is an integral membrane protein, we suggest that these quantitative results might correspond to APP fragments. This hypothesis was confirmed by WB analysis. Two bands corresponding to putative APP fragments were detected, a faint band between 48 kDa and 63 kDa and an intense band at 25 kDa that might correspond, respectively, to amyloid fragments such as Aβ40 and Aβ42 (125) and c-terminal fragments from APP resultant from proteolytic processing (126). This APP fragment (25 KDa) was highly abundant in vitreous from AMD patients, pointing to a potential biomarker of neurodegenerative vitreoretinal diseases. Associated with amyloidosis, CST3 was also found upregulated in AMD compared to DR, but higher levels were found in RRD. CST3 is a potent inhibitor of lysosomal and extracellular cysteine proteinases ubiquitously expressed by all mammalian tissues and present in all body fluids. In the eye, it is particularly abundant in RPE (127). Mutations in CST3 genes were associated with an increased risk of developing nAMD (128) and hereditary cerebral haemorrhage with amyloidosis (129). Mutant variants of CST3 form deposits with APP peptides in senile plaques and arteriolar walls in the brain of AD patients, suggesting a role in amyloidosis (127, 128). On the other hand, it has been suggested the involvement of CST3 in several neuroprotective mechanisms by inhibition of cysteine proteases and induction of autophagy, induction of neurogenesis, and inhibition of oligomerization and amyloid fibril formation (130). Another interesting outcome from our study was the high levels of CST3 found in DRR/PVR, which was previously reported by Yu and co-workers (44). To our knowledge, there are no studies regarding the role of CST3 in RRD/PVR, but some evidence indicates that it inhibits the epithelial-mesenchymal transition, a clinical feature of PVR that occurs in RPE cells, in mammary epithelial cells (131).

## Conclusion

The characterization of the vitreous humor proteome is essential for the elucidation of the molecular mechanisms underlying ocular pathologies. Nevertheless, most potential biomarkers described to date have not been validated in a large cohort, limiting their utility in clinical practice. We have applied an LFQ-based method to analyze the vitreous proteome in PDR and AMD compared to ERM. Our findings agree with previous results and reinforce the involvement of complement and coagulation cascades in the pathogenesis of PDR and nAMD. However, our findings suggest that these proteins are not specific biomarkers of any of these pathologies, but suitable predictors instead of their progression to a proliferative etiology. In addition, a significant number of adhesion molecules, nervous system development proteins, lysosomal proteins, and ECM components were found up-regulated in dry AMD and ERM, reinforcing the neurodegenerative nature of these pathologies. This indicates that the use of vitreous from patients with ERM (or other pathologies such as macular holes) as surrogate control should be taken carefully. Although functional analysis did not highlight proteins related to angiogenesis, the downregulation of anti-angiogenic factors such as OPTC and PEDF in PDR and AMD might suggest that the vitreous humor in these pathologies could be being transformed in an environment prone to angiogenic processes. An interesting outcome of our results is the central role of APP in neurodegeneration as it integrates multiple pathways, emphasizing the multifactorial nature of these diseases. Our analysis provided a list of biomarkers with the potential to discriminate between several vitreoretinal diseases, including DR/PDR, AMD, DDR/PVR, and ERM. According to ROC curves, complement and coagulation components (C2 and prothrombin), acute-phase mediators (AACT), adhesion molecules (e.g., myocilin, LGALS3BP), ECM component (OPTC), and neurodegeneration biomarkers (APP and amyloid-like protein 2) are the most efficient discriminators between different disease groups. In conclusion, our study illuminates some of the mechanisms underlying PDR and AMD and provides potential biomarkers in vitreous. These proteins could be assessed in samples obtained as part of the clinical routine for the prognosis of the disease and the response to treatment. In addition, they could be potential target candidates for the development of new pharmaceutical drugs.

## Data availability statement

The datasets presented in this study can be found in online repositories. The names of the repository/repositories and accession number(s) can be found in the article/**Supplementary Material**.

## Ethics statement

The studies involving human participants were reviewed and approved by Affiliation: Ophthalmology Service of Leiria-Pombal Hospital, Portugal. Code of protocol approved by the hospital ethics committee: CHL-15481. The patients/participants provided their written informed consent to participate in this study.

## Author contributions

The experiments were conceptualized and designed by FS, SC, CT, and AP. JS performed pars plana vitrectomy and assisted JM in the storage of vitreous samples and collection of information about the patients. FS, SC, CC, and AP conduct the experiments and analyzed the data. FS and SC were responsible for data curation. FS prepared the original draft and SC, JM, LP, CT, and AP reviewed the manuscript. This worked was supervised by LP, CT, and AP. All authors contributed to the article and approved the submitted version.

## Glossary

**Table:**

A1AT	Alpha-1-antitrypsin
AACT	Alpha-1-antichymotrypsin
AMD	Age-Related Macular Degeneration
APLP2	Amyloid-like protein 2
APP	Beta-Amyloid
BCAN	Brevican core protein
CADH2	neural cadherin
CATZ	Cathepsin Z
CE	Collision energy
CFH	Complement factor H
CHGA	Chromogranin-A
CLU	Clusterin
C2	Complement C2
C5	Complement C5
C6	Complement C6
C8B	Component C8 beta chain
C9	Complement C9
CSTC	Cystatin-C
CSTN1	Calsyntenin-1
DP	Declustering Potential
DR	Diabetic Retinopathy
ECM	Extracellular Matrix
ERM	Epiretinal Membranes
F2	Prothrombin
F9	Coagulation Factor IX
GNS	N-acetylglucosamine-6-sulfatase
IDA	Information-dependent acquisition
IGFBPs	Insulin-like Growth Factor Binding Proteins
IGFs	Insulin-like Growth Factor
IHT	Interface heater temperature
ITIH3	Inter-alpha-trypsin Inhibitor Heavy Chain H3
LGALS3BP	Galectin-3-binding Protein
MNV	Macular neovascularization
MRM	Multiple Reaction Monitoring
nAMD	“Wet” or Neovascular AMD
NCAN	Neurocan core protein
NRX3A	Neurexin-3
OPTC	Opticin
OSTP	Osteopontin
PDR	Proliferative Diabetic Retinopathy
PEDF	Pigment Epithelium-Derived Factor
PVR	Proliferative Vitreoretinopathy
ROC	Receiver Operating Characteristic
RRD	Rhegmatogenous Retinal Detachment
SOD3	Extracellular Superoxide Dismutase [Cu-Zn]
SPON1	Spondin-1
TIMP1	Metalloproteinase Inhibitor 1
TIMP2	Metalloproteinase Inhibitor 2
TNR	Tenascin-R
VEGF	Vascular Endothelial Growth Factor
XLRS1	Retinoschisin
