# Supplementary material for: Proteomics profiling of vitreous humor reveals complement and coagulation components, adhesion factors, and neurodegeneration markers as discriminatory biomarkers of vitreoretinal eye diseases
Source: Front Immunol. 2023 Feb 16;14:1107295. doi: 10.3389/fimmu.2023.1107295 (PMC9978817; doi:10.3389/fimmu.2023.1107295)

Supplementary Figures

Supplementary Figure 1. MultiScatter plots comparing the correlation (Pearson) of all the vitreous samples analyzed in the label-free experiment before and after the removal of VH 219 from the statistical analysis

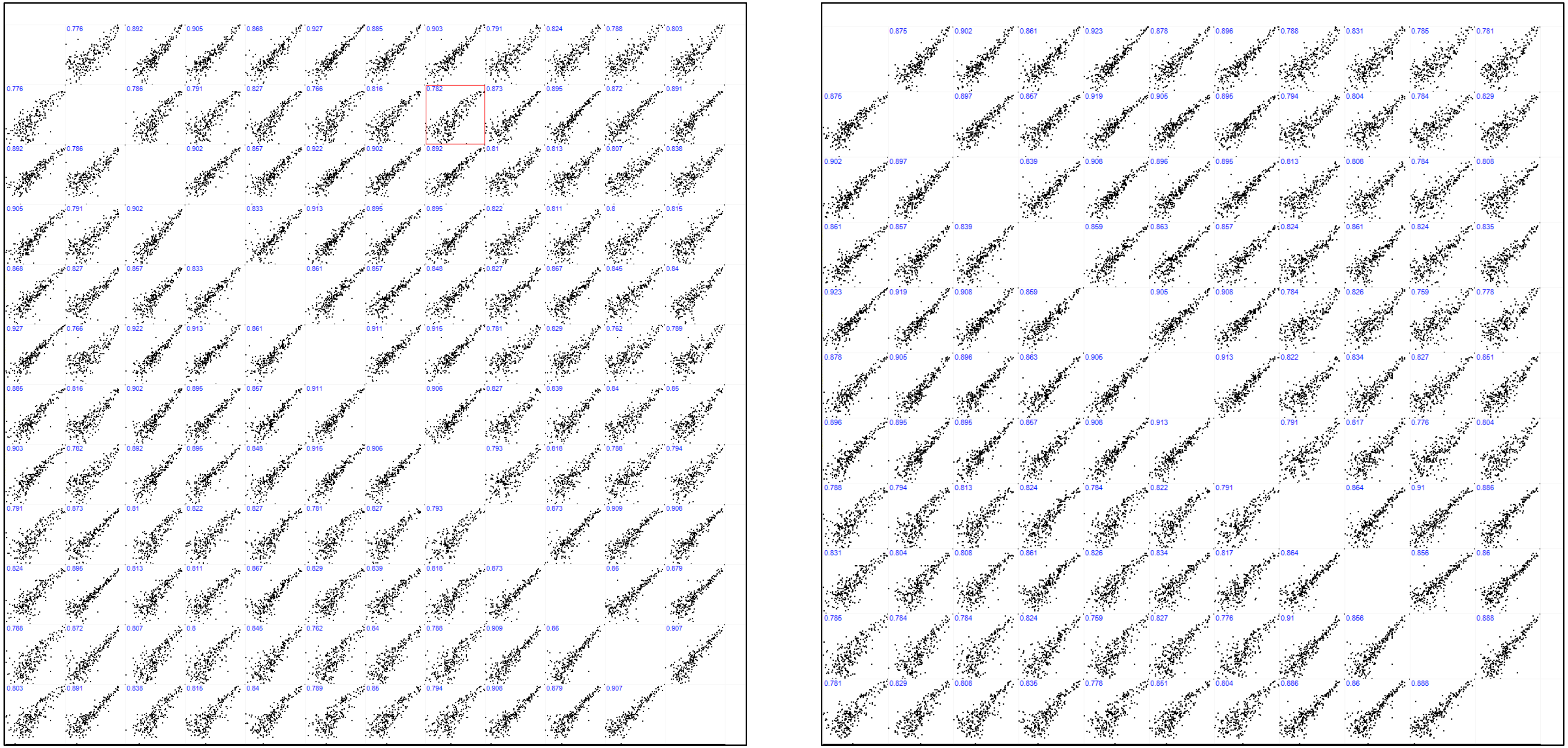

Supplementary Figure 2. Comparison of normalized intensities from the label-free experiment with the results obtained in western blot analysis of (A) Chromogranin-A (CGMA) and (B) Metalloproteinase inhibitor 2 (TIMP2). Data are presented as mean ± SD with q-values of <0.05 (*) and q-value<0.01 (**).

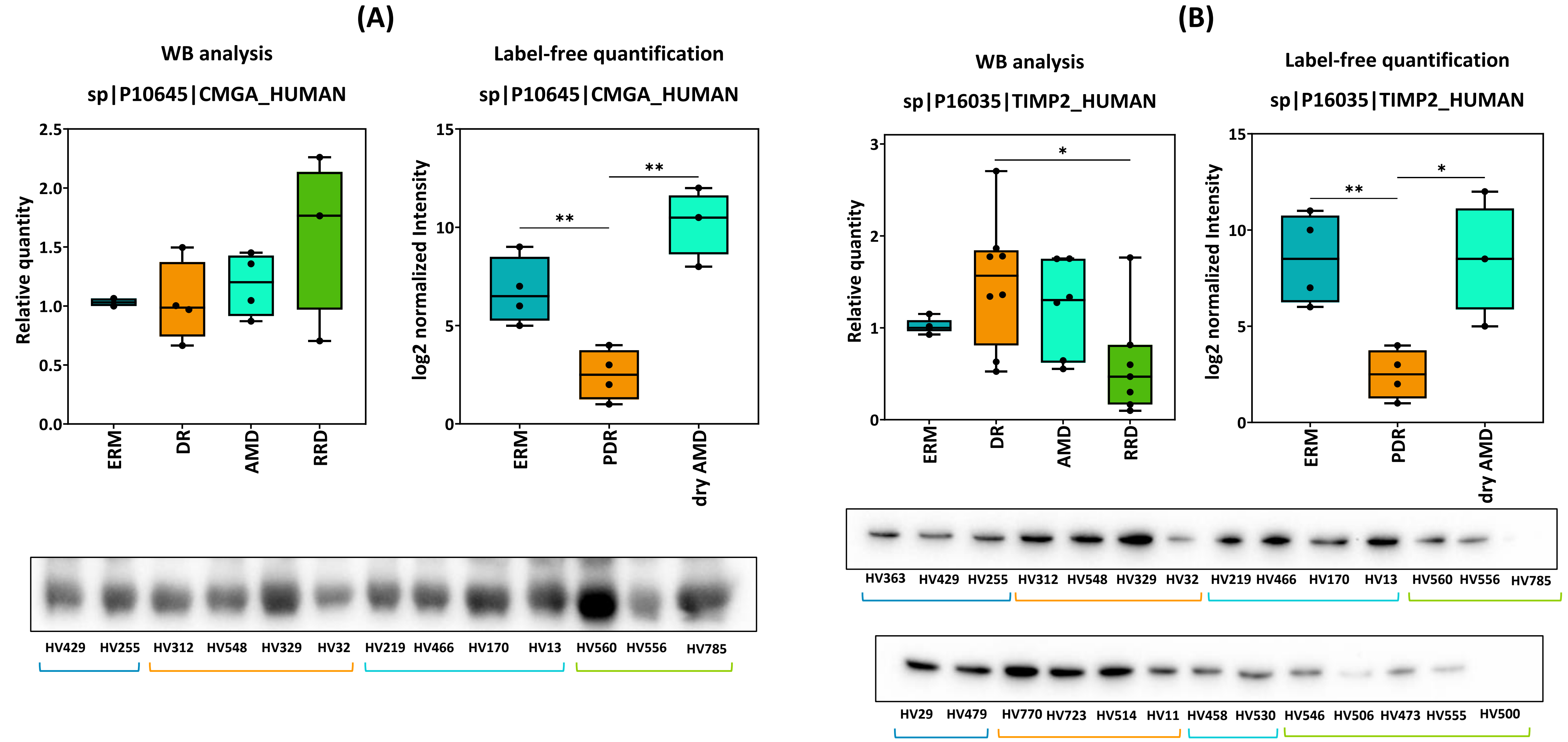

Supplementary Figure 3.1. Results obtained in the analysis of beta-amyloid (APP) by western blot.

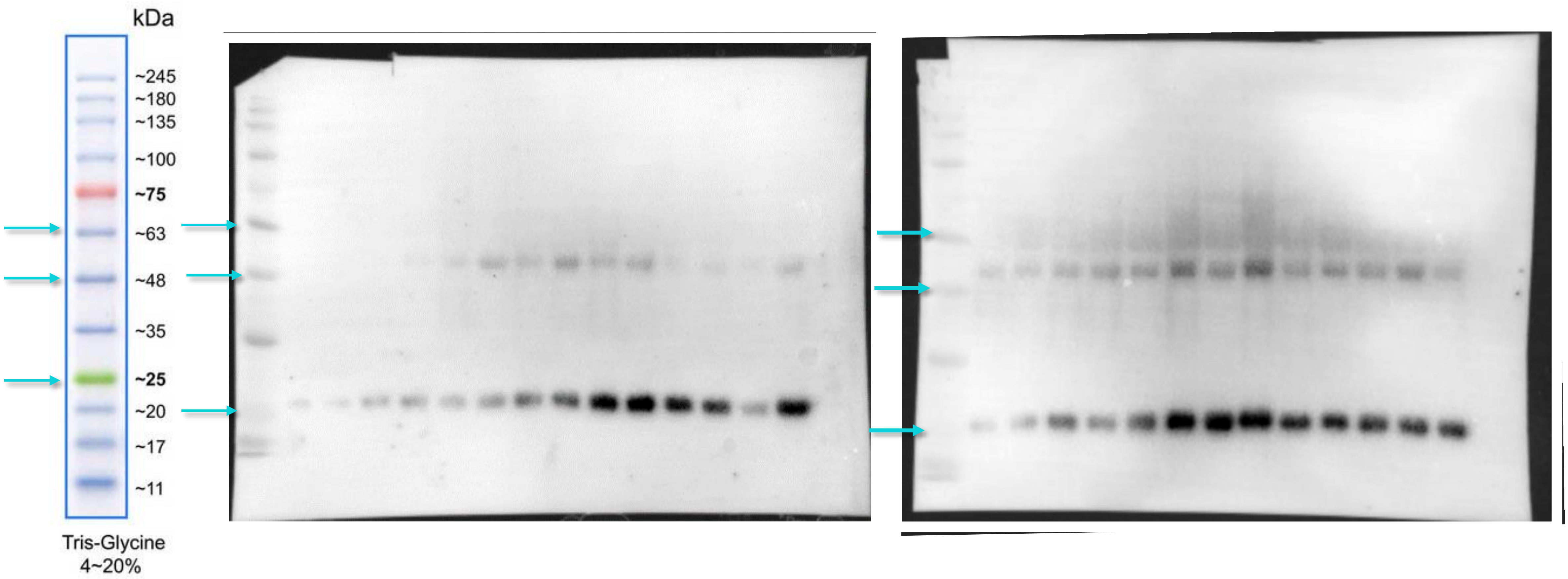

Supplementary Figure 3.2. Results obtained in the analysis of cystatin-C (CSTC) by western blot.

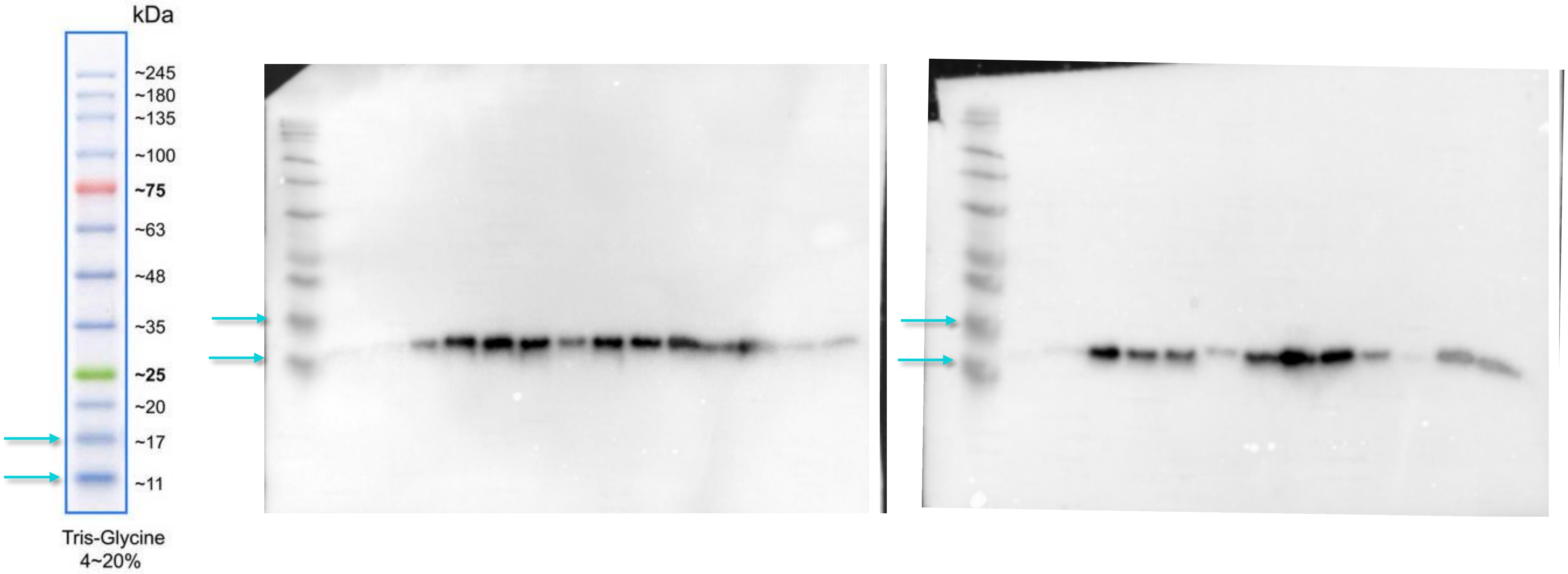

Supplementary Figure 4.1. Results of the multivariate exploratory receiver operating characteristic (ROC) analysis for assess the accuracy of the panel of a panel of fifteen candidate biomarkers in the classification of Diabetic Retinopathy vs Epiretinal Membranes.

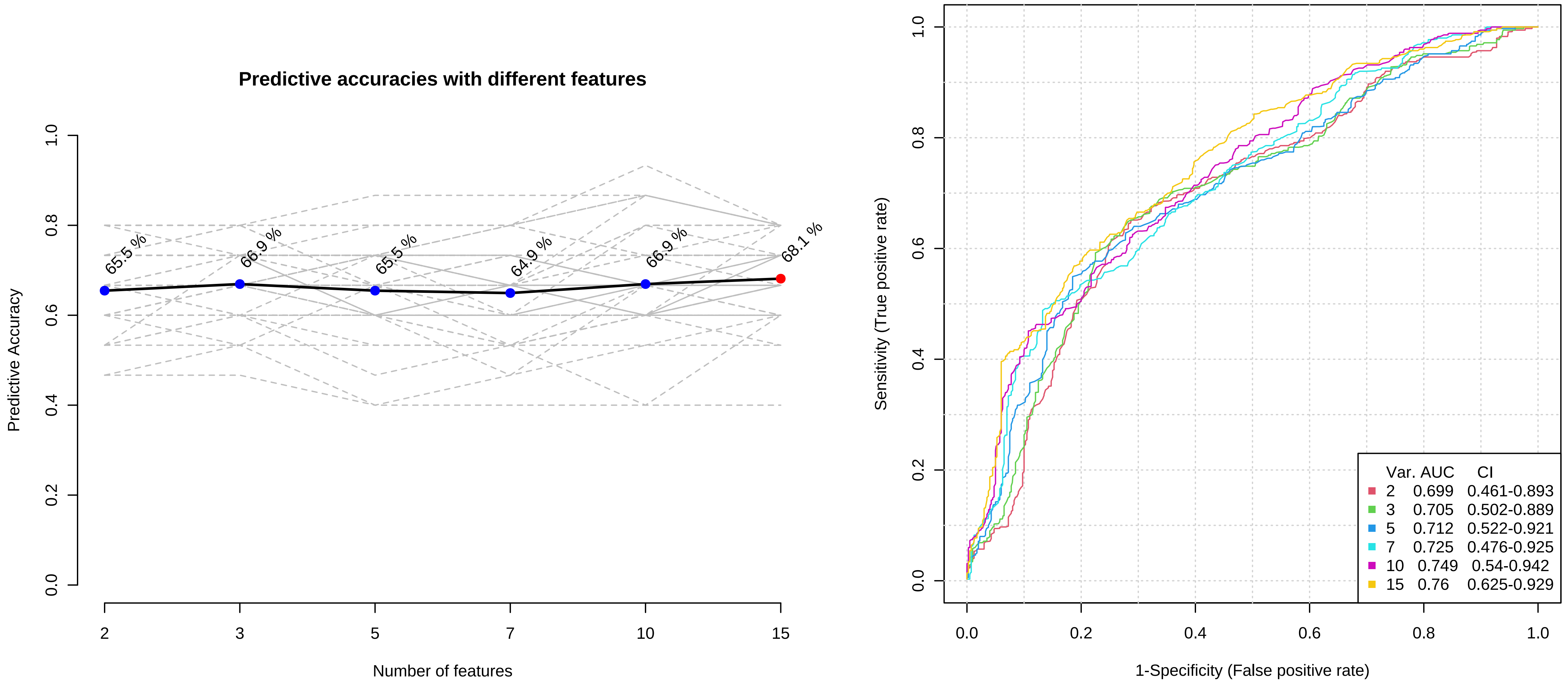

Supplementary Figure 4.2. Results of the multivariate exploratory receiver operating characteristic (ROC) analysis for assess the accuracy of the panel of a panel of fifteen candidate biomarkers in the classification of Diabetic Retinopathy vs Age-related Macular Degeneration.

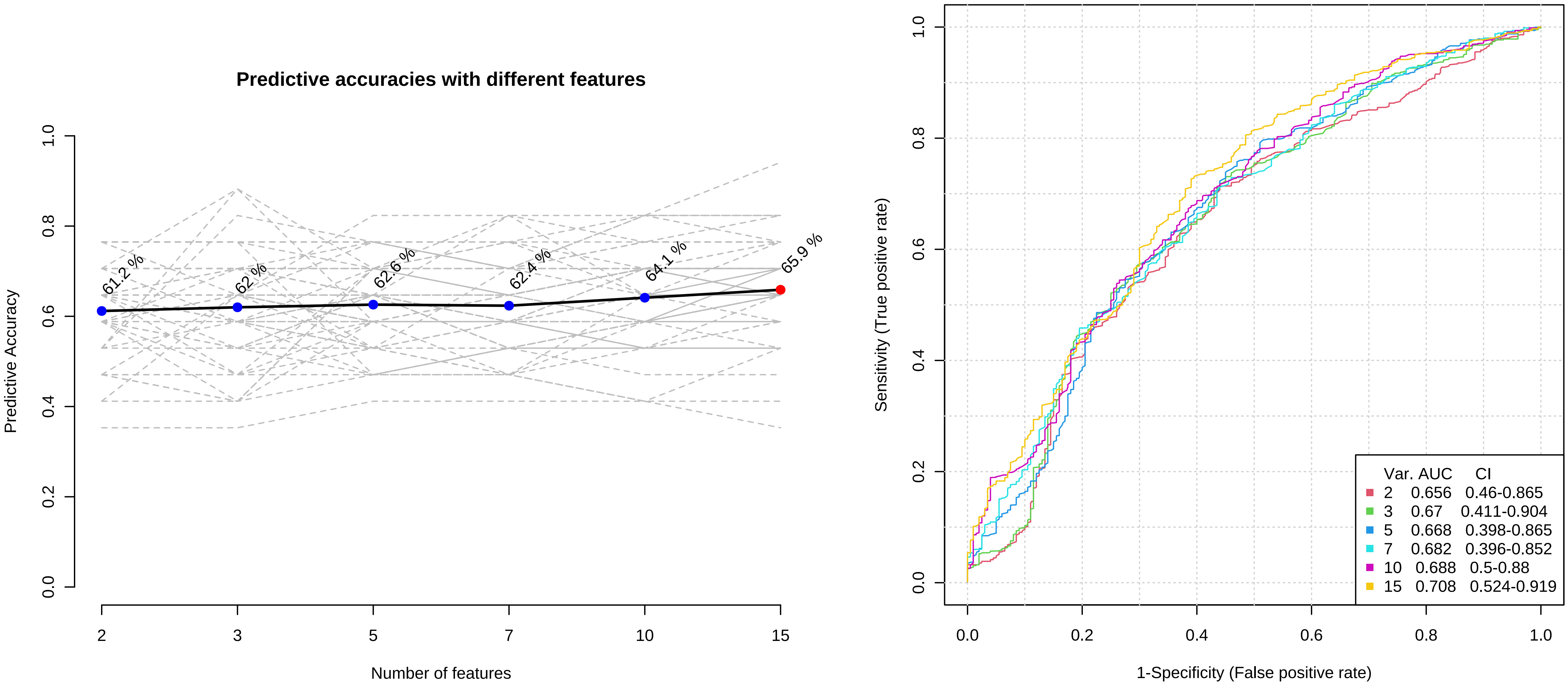

Supplementary Figure 4.3. Results of the multivariate exploratory receiver operating characteristic (ROC) analysis for assess the accuracy of the panel of a panel of fifteen candidate biomarkers in the classification of Diabetic Retinopathy vs Rhegmatogenous Retinal Detachment.

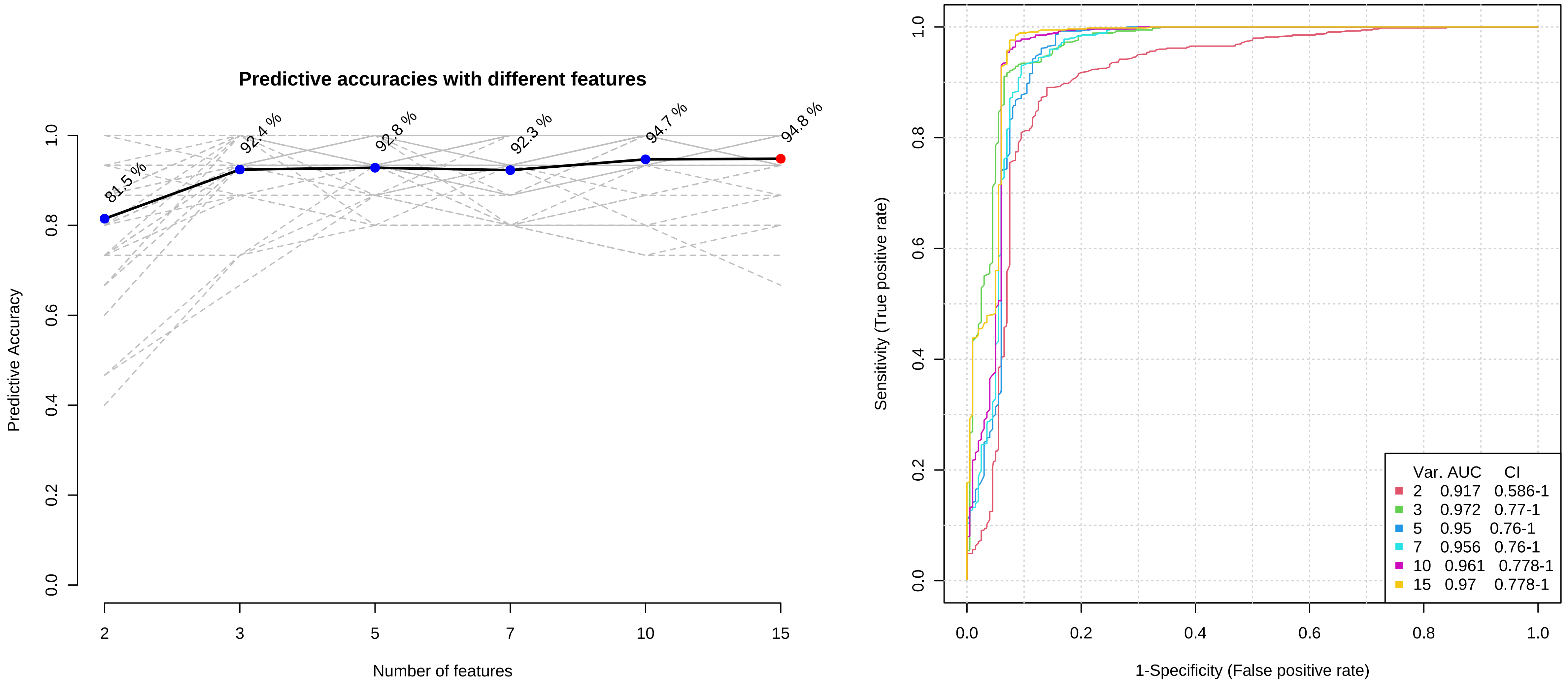

Supplementary Figure 4.4. Results of the multivariate exploratory receiver operating characteristic (ROC) analysis for assess the accuracy of the panel of a panel of fifteen candidate biomarkers in the classification of Age-related Macular Degeneration vs Epiretinal Membranes.

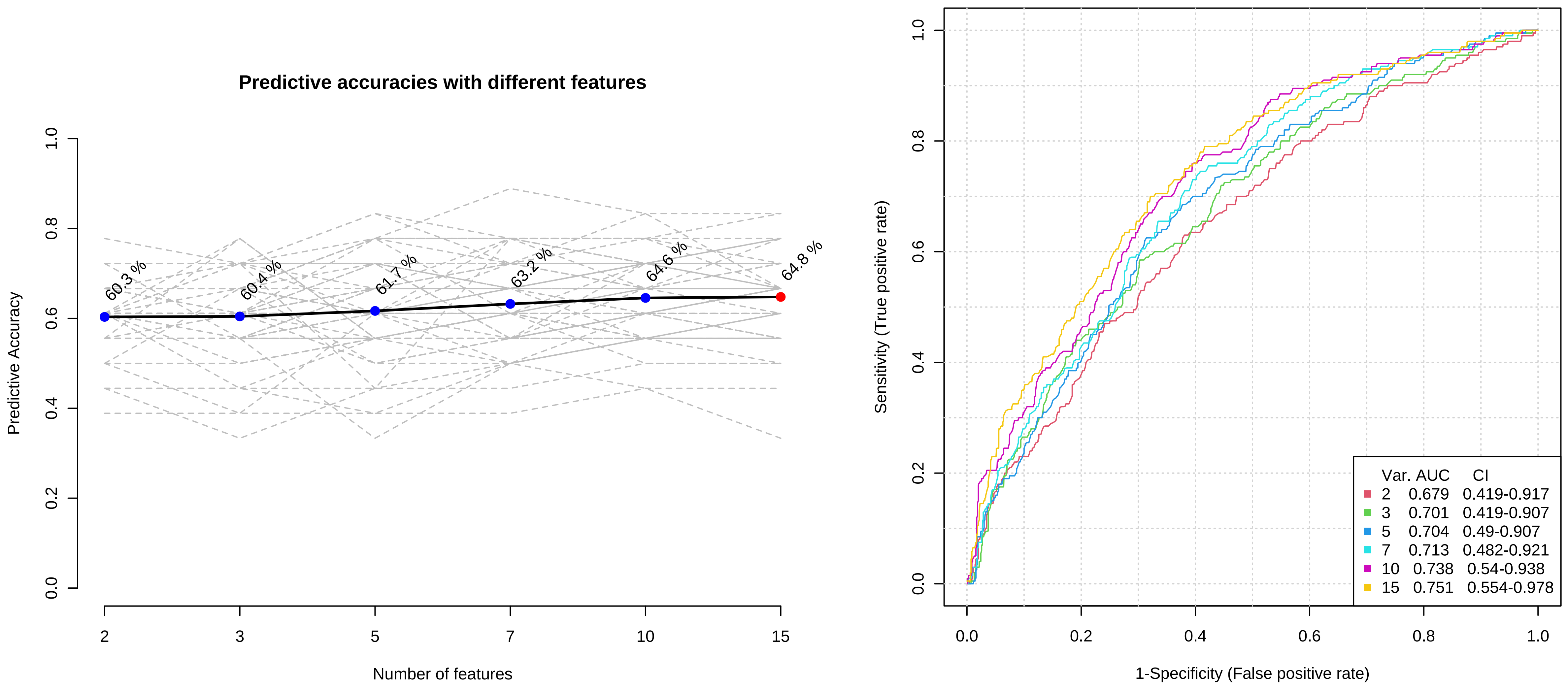

Supplementary Figure 4.5. Results of the multivariate exploratory receiver operating characteristic (ROC) analysis for assess the accuracy of the panel of a panel of fifteen candidate biomarkers in the classification of Age-related Macular Degeneration vs Rhegmatogenous Retinal Detachment.

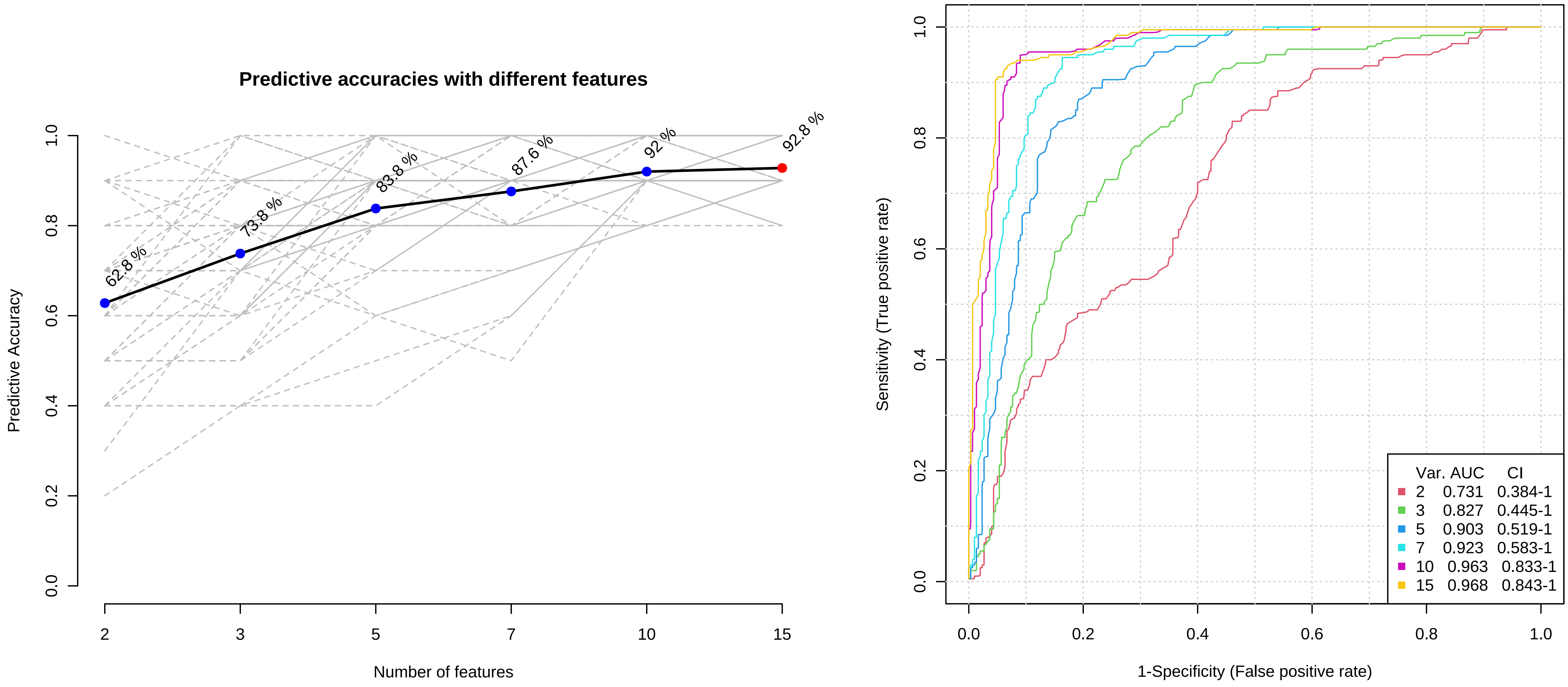

Supplementary Figure 4.6. Results of the multivariate exploratory receiver operating characteristic (ROC) analysis for assess the accuracy of the panel of a panel of fifteen candidate biomarkers in the classification of Rhegmatogenous Retinal Detachment vs Epiretinal Membranes.

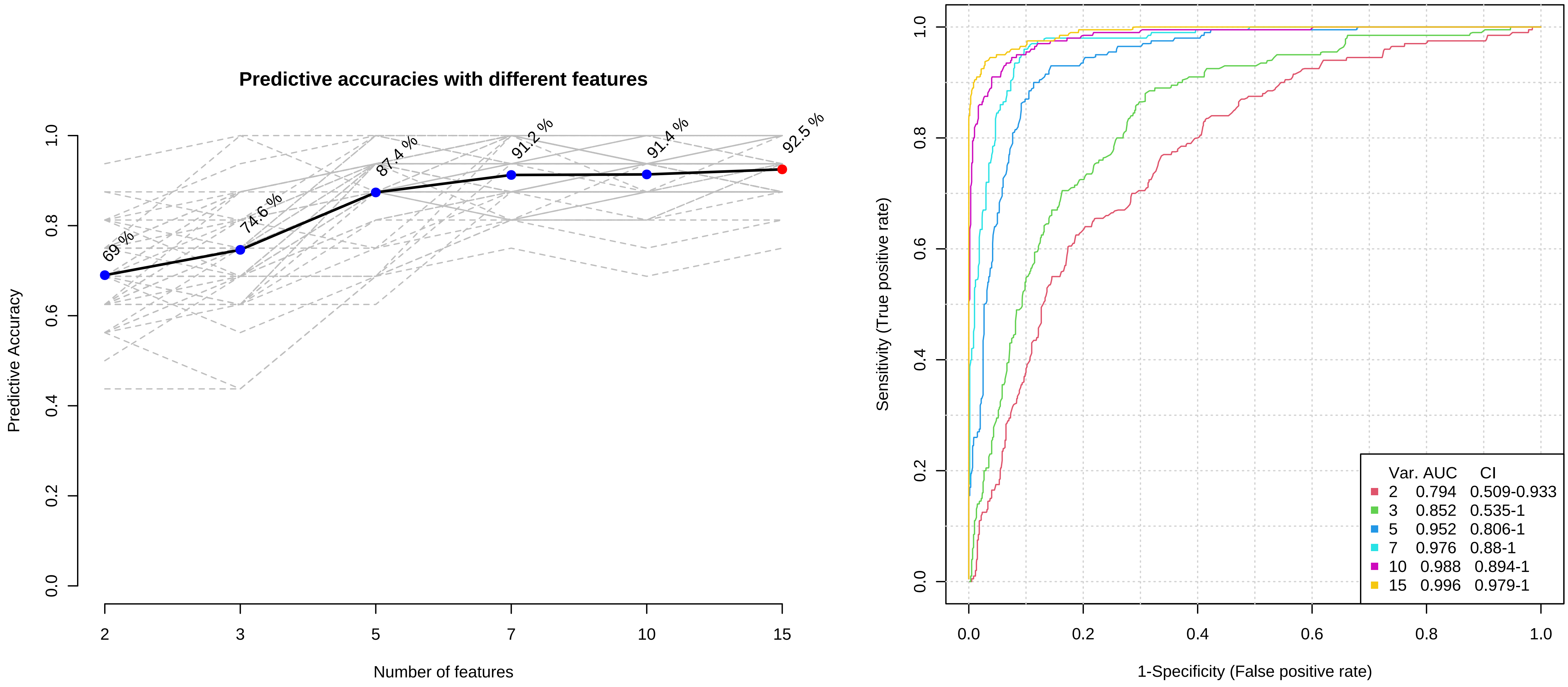

Supplement: Supplementary file 1 [file DataSheet_1.pdf]
